# Synthesis of Novel *N*-Methylmorpholine-Substituted Benzimidazolium Salts as Potential α-Glucosidase Inhibitors

**DOI:** 10.3390/molecules27186012

**Published:** 2022-09-15

**Authors:** Imran Ahmad Khan, Furqan Ahmad Saddique, Sana Aslam, Usman Ali Ashfaq, Matloob Ahmad, Sami A. Al-Hussain, Magdi E. A. Zaki

**Affiliations:** 1 Department of Chemistry, Government College University, Faisalabad 38000, Pakistan; 2 Department of Chemistry, Government College Women University, Faisalabad 38000, Pakistan; 3 Department of Bioinformatics and Biotechnology, Government College University, Faisalabad 38000, Pakistan; 4 Department of Chemistry, Faculty of Science, Imam Mohammad Ibn Saud Islamic University (IMSIU), Riyadh 11623, Saudi Arabia

**Keywords:** benzimidazole, morpholine, benzimidazolium salts, molecular docking, α-glucosidase inhibition, antidiabetic studies

## Abstract

The α-glucosidase enzyme, located in the brush border of the small intestine, is responsible for overall glycemic control in the body. It hydrolyses the 1,4-linkage in the carbohydrates to form blood-absorbable monosaccharides that ultimately increase the blood glucose level. α-Glucosidase inhibitors (AGIs) can reduce hydrolytic activity and help to control type 2 diabetes. Aiming to achieve this, a novel series of 1-benzyl-3-((2-substitutedphenyl)amino)-2-oxoethyl)-2-(morpholinomethyl)-1*H*-benzimidazol-3-ium chloride was synthesized and screened for its α-glucosidase inhibitory potential. Compounds **5d**, **5f**, **5g**, **5h** and **5k** exhibited better α-glucosidase inhibitions compared to the standard drug (acarbose IC_50_ = 58.8 ± 0.012 µM) with IC_50_ values of 15 ± 0.030, 19 ± 0.060, 25 ± 0.106, 21 ± 0.07 and 26 ± 0.035 µM, respectively. Furthermore, the molecular docking studies explored the mechanism of enzyme inhibitions by different 1,2,3-trisubstituted benzimidazolium salts via significant ligand–receptor interactions.

## 1. Introduction

Diabetes, or diabetic mellitus (DM), is a chronic metabolic disease characterized by unusually high levels of plasma glucose and is associated with severe health concerns [1,2]. In 2016, the WHO reported that every 11th person is affected by diabetic mellitus globally, and it will be the seventh highest cause of death until 2030 [3]. Mechanistically, diabetes can be either of three types: type 1 diabetes (T1D), type 2 diabetes (T2D) and gestational diabetes (GD). Type 1 diabetes is an autoimmune disorder, developed by the destruction of insulin-producing β-cells in the endocrine pancreas [4]. As the β-cell population drops, the absolute deficiency of available insulin to maintain the normal blood glucose level develops, and type 1 diabetes is mainly diagnosed in children and young adults [5,6]. Metabolic irregularity characterized by insulin resistance can lead to the development of type 2 diabetes. Type 2 diabetes is accompanied with pathophysiological changes which cause disturbances in normal glucose homeostasis. In type-2-diabetic patients, continuous insulin resistance in the body leads to the formation of fasting hyperglycemia, and ultimately, the further suppression of pancreatic β-cells causes chronic hyperglycemia to develop [7]. An elevated blood glucose level after a meal, often above 200 mg/dL, referred as postprandial hyperglycemia, is a direct factor for atherosclerosis, cardiovascular disorder, hypertension, stroke, kidney failure, retinopathy, neuropathy, lipid metabolism disorder and other diabetic complications [8,9]. Gestational diabetes, as is obvious from the name, is diagnosed in pregnant women and is linked with severe medical problems for mother and offspring [10].

α-Glucosidase is an axo-enzyme, present in the small intestine, and is responsible for the production of glucose by catalyzing the breakdown of starch and dietary carbohydrates [11]. Therefore, the inhibition of α-glucosidase enzymes is a vital approach to preventing postprandial hyperglycemia in type-2-diabetic patients. Various AGIs, including FDA-approved AGIs such as acarbose, miglitol and voglibose, perform their action by decreasing the carbohydrate digestion in the body [12]. However, the prolonged use of these drugs is associated with some adverse side effects such as diarrhea, flatulence, abdominal pain and gastrointestinal infections. Therefore, there is an urgent need to explore new and effective AGIs.

**Figure 1:**
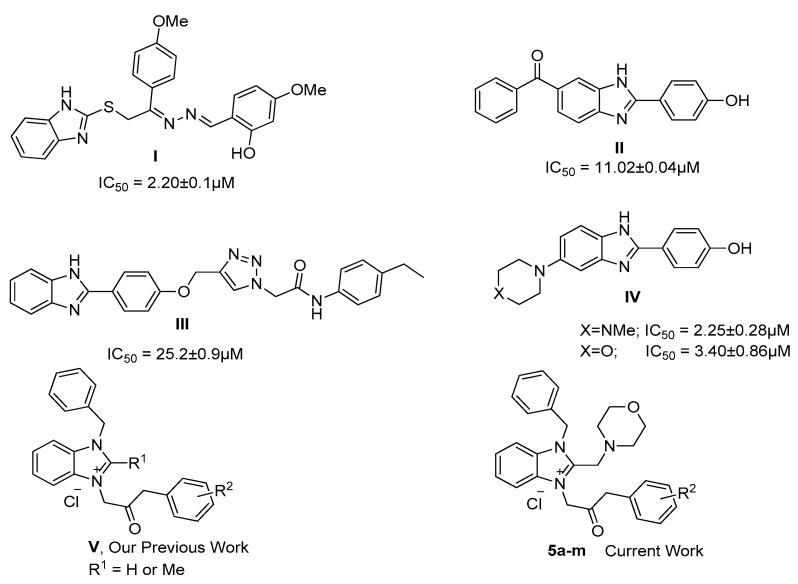
Chemical structures of benzimidazole-based α-glucosidase inhibitors.

Benzimidazole, bearing two endocyclic nitrogen atoms, has strong potential as a therapeutic agent. The structural similarity of benzimidazole with a purine base makes it an effective ligand for interaction with biopolymers present in the living systems [13]. Either alone or given in combination with other drugs, it has established its repute as anticancer [14], anti-inflammatory [15], antimalarial [16], antidiabetic [17], anti-HIV [18], antimicrobial [19], antidepressant [20] and as an enzyme-inhibiting drug [21]. In the context of α-glucosidase inhibitors (AGIs), benzimidazole scaffolds are well recognized in the literature as AGIs. These scaffolds include benzimidazole-bearing Schiff bases (**I,** Figure 1) [12], benzoylarylbenzimidazoles (**II,** Figure 1) [22], benzimidazole-1,2,3-triazole hybrids (**III,** Figure 1) [23], structural hybrids with piperazine and morpholine (**IV,** Figure 1) [24], etc.

Currently, benzimidazole-based quaternary salts (also known as benzimidazolium salts) have gained interest due to their strong inhibitory potential against cholinesterase [25], α-glucosidase [26] and paraoxanse 1 enzymes [27]. Benzimidazolium-based organic cages were recently reported as antimicrobial agents [28]. Previously, our research group successfully reported amide-functionalized benzimidazolium salts as α-glucosidase inhibitors [29], and it was shown that C^2^ substitution on benzimidazole has a key role in determining the α-glucosidase inhibitory potential. Motivated by previous observations, herein, an attempt was made to synthesize biologically active hybrid molecules bearing benzimidazole and morpholine heterocycles where the C^2^ position of the benzimidazolium ring was incorporated by a pharmacologically active *N*-methyl morpholine (NMM) scaffold. Finally, all of the target molecules were investigated for their in vitro and in silico α-glucosidase inhibitory potential.

## 2. Results and Discussion

### 2.1. Chemistry

**Scheme 1:**
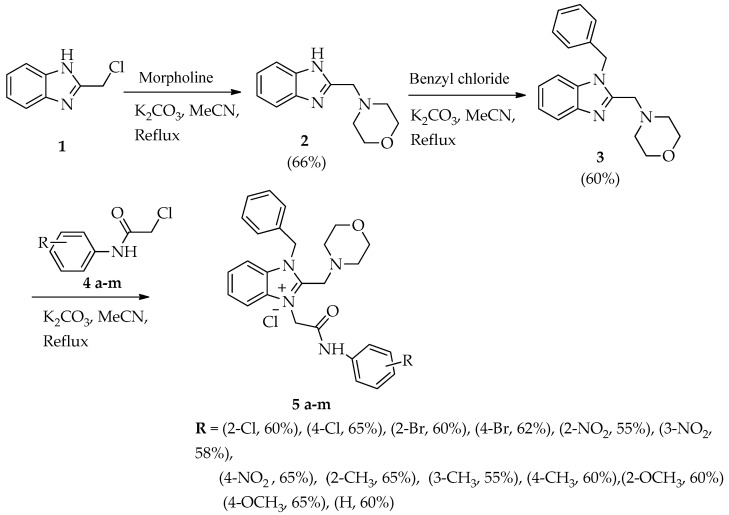
Synthesis of targeted *N*-methylmorpholine-substituted benzimidazolium salts.

The general route for the synthesis of *N*-methylmorpholine-functionalized benzimidazolium salts is depicted in Scheme 1. Initially, a substitution reaction between 2-(chloromethyl)-1H-benzimidazole **1** and morpholine in the presence of potassium carbonate in acetonitrile afforded 4-((1H-benzimidazol-2-yl)methyl)morpholine **2**. The *N*-alkylation of 4-((1H-benzimidazol-2-yl)methyl)morpholine **2** with benzyl chloride provided 4-((1-benzyl-1H-benzo[d]imidazol-2-yl)methyl)morpholine intermediate **3**. Finally, the quaternization of 1,2-disubstituted benzimidazole intermediate **3** with a variety of 2-chloro-*N*-arylacetamides **4a**–**m** yielded the 1-benzyl-2-(morpholinomethyl)-3-(2-oxo-2-(substitutedphenylamino)ethyl)-1H-benzo[d]imidazol-3-ium chlorides **5a**–**m** in moderate yields (55–65%). Each reaction was monitored by TLC using a mixture of chloroform and methanol in a 4:1 ratio. All of the synthesized salts were soluble in polar solvents (methanol, chloroform, etc.).

### 2.2. Characterization

The structures of these morpholine-based benzimidazolium salts **5a**–**m** were well characterized with the help of ^1^H NMR, ^13^C NMR spectroscopic and HRMS techniques (Supplementary Data File). In the ^1^H NMR spectra of all the benzimidazolium salts, a characteristic sharp singlet observed in the range of 10–12.01 ppm was assigned to an NH proton of amide functionality. Morpholine protons were observed in the form of two broad singlets at 2.34 and 3.95 ppm. Chemical shift values in the range of 2.25–2.33 ppm were assigned to methyl protons present in **5h**, **5i** and **5j** benzimidazolium salts. A broad singlet at 3.95–4.38 ppm characterized the protons of the methylene group linked to morpholine moiety. Another downfield singlet in the range of 5.59–5.82 ppm indicated the presence of benzylic (-CH_2_-) protons. Similarly, a singlet in the range of 5.98–5.99 ppm was assigned to methylene protons of acetamide functionality. Aromatic protons were observed in the aromatic region (6.91–8.15 ppm). In the case of ^13^C NMR spectra, the carbonyl carbons (C=O) were characterized by sharp peaks in the range of 163.6–165.6 ppm. The spectroscopic data of products were consistent with the literature data [29] regarding closely related structures.

### 2.3. In Silico α-Glucosidase Inhibition

The structural modification of lead molecules has been an effective approach for drug discovery. However, this practice is costly and time-consuming for researchers. However, these weaknesses can be overcome by adopting in silico techniques. A molecular docking study is an in silico technique used for the identification of biologically effective templates prior to their synthesis. Molecular docking helps towards targeted synthetic work which is much needed in the modern era. A variety of ligands were docked into the selective pocket of a receptor enzyme, which provided valuable information about the interaction modes and inhibition mechanisms of the docked ligands [30,31]. Interaction studies are further utilized by medicinal chemists to devise layouts for potent molecules via structural modifications [30,31,32]. Herein, we executed molecular docking studies of potent *N*-methylmorpholine-substituted benzimidazolium salts for the inhibition of the α-glucosidase enzyme (PDB ID: 2QMJ) using Molecular Orbital Environment (MOE) software (Montreal, Canada, Ver.2014.0901). The molecular docking results were analyzed on the basis of binding energy, rmsd values and important interactions with the targeted enzyme, which helped us to categorize the screened derivatives [33].

**Figure 2:**
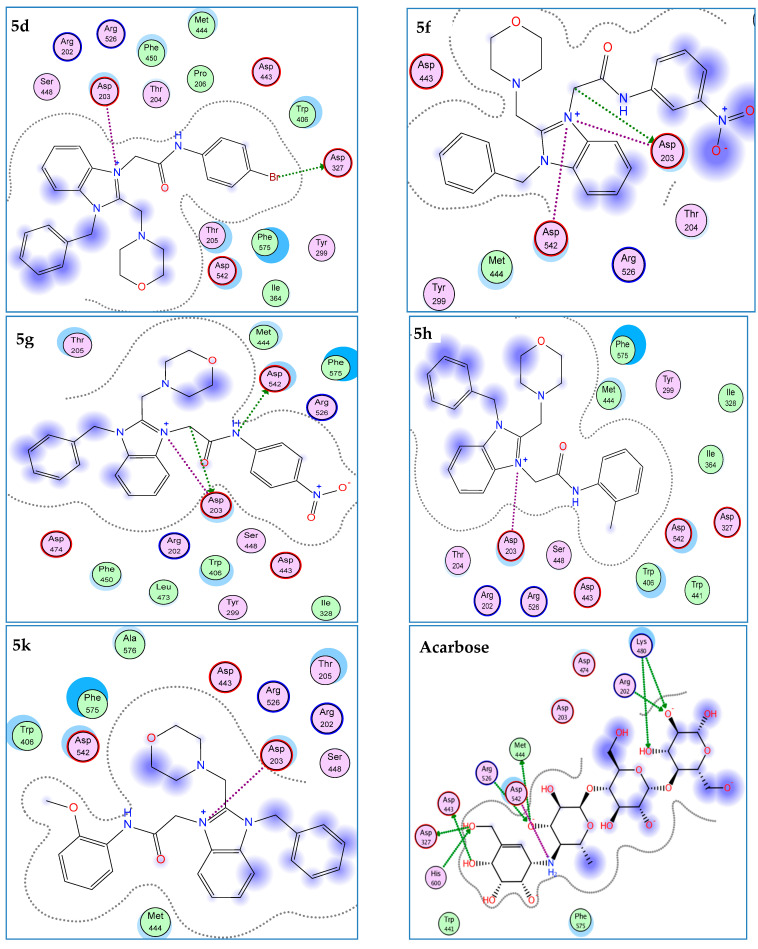
Two-dimensional interaction modes of potent compounds (**5d**, **5f**, **5g**, **5g** and **5k**) and standard drug, acarbose. The blue color indicates ligand exposure, and the green-colored dotted lines indicate the interactions of receptor enzyme with ligands.

**Figure 3:**
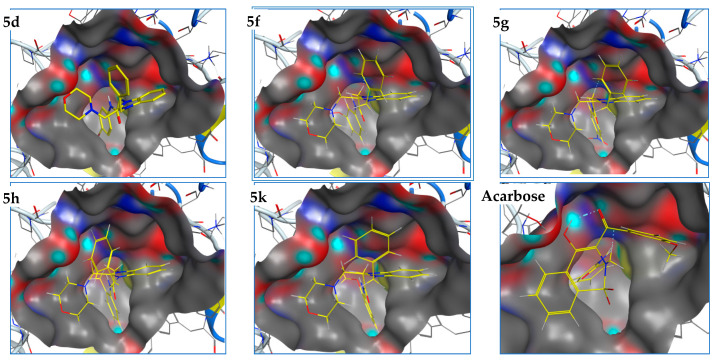
Three-dimensional interaction modes of active compounds (**5d**, **5f**, **5g**, **5h** and **5k**) and standard drug, acarbose. Color portion shows the pocket selected, and red portions indicate the ligand exposure points.

All the potent compounds exhibited good interactions with the selected residues, Asp203, Asp542, Asp327, His600 and Arg526 (Figure 2 and Figure 3). In-addition, the derivatives **5d**, **5f**, **5g**, **5h** and **5k** were observed to have good binding scores and low rmsd values (Table 1). The docking scores of these compounds were found in the range of −12.1 to −13.15 Kcal/mol, while rsmsd values were observed to be less than 2 Å [33]. The docking scores of these compounds were found to be close to the docking score of the standard drug, acarbose (−13.87 Kcal/mol) (Table 1). The ligands **5d**, **5f**, **5g**, **5h** and **5k** preferably interacted with Asp203, Asp542 and Asp327 residues among the selected residues. Hence, bindings with these residues were found to be important and responsible for the enzyme inhibition mechanism of screened ligands. Previously, Nakamura et al. conducted a detailed molecular docking study of salacinol derivatives against the α-glucosidase enzyme. They concluded that the residues Asp203, Asp542 and Asp327 are involved in the enzyme inhibitory potential of salacinol derivatives [34]. In 2020, Promyos et al. carried out an in silico analysis of various anthocyanins and anthocyanidins against the α-glucosidase enzyme. These derivatives also showed significant interactions with Asp203, Asp542 and Asp327 residues [35]. In another report, Nursamsiar et al. presented derivatives of aglycone of curculigoside A as good inhibitors of the α-glucosidase enzyme. These templates also exhibited significant interactions with Asp203, Asp327 and Asp542 residues [36]. Similarly, various 1,2-benzothiazine-*N*-arylacetamides were found to inhibit the α-glucosidase enzyme by interacting with Asp203, Asp542 and Asp327 residues [37,38]. Xenthone derivatives demonstrated their in silico inhibitory potential with the α-glucosidase enzyme via blocking the Asp203 and Asp542 residues [39]. In another in silico study, 8-c-Ascorbyl (-)-epigallocatechin (present in black tea) showed binding interactions with the Asp203 and Asp542 residues [40].

The two-dimensional molecular docking maps showed the binding modes of screened ligands (Figure 2). Compound **5d** showed interactions with Asp327 and Asp203. A dipole–dipole interaction between Asp327 and the bromo group of ligand **5d** and a pi–cation interaction between Asp203 and the iminium nitrogen of ligand **5d** were observed. This ligand interacted with Asp327 and Asp203 using its bromo group and iminium nitrogen, respectively. A good binding score of −13.15 Kcal/mole and a low rmsd value of 1.01 Å were observed for this ligand. Similarly, the derivative **5f** exhibited three interactions. Hydrogen bonding was observed between residue Asp542 and the –NH group of ligand **5f**. The residue Asp203 interacted with the methylene and iminium nitrogen of the ligand **5f** via dipole–dipole and cation–pi interactions, respectively. These bindings were found responsible for the low docking score (−13.10 Kcal/mol) and rmsd value (1.15 Å) of this derivative. Derivative **5g** also showed a good docking score of −12.01 Kcal/mol and a low rmsd value of 1.45 Å, which were due to interactions of this ligand with Asp203 and Asp542. Asp203 showed a cation–pi interaction with the iminium nitrogen and a dipole–dipole interaction with the methylene group, while Asp542 formed a hydrogen bond with the –NH group of the compound **5g**. The ligands **5h** and **5k** exhibited the same type of interaction modes. Both these ligands formed a cation–pi interaction with Asp203 using their iminium nitrogen atoms. The ligands **5h** and **5k** were found to have docking scores of −12.75 and −13.87 Kcal/mol, respectively. Similarly, the rmsd values of these ligands were found to be 1.19 and 1.38 Å, respectively. The standard drug, acarbose, also showed a good docking score of −13.87 Kcal/mol in a re-dock experiment; however, its rmsd value was found to be high (2 Å).

The molecular docking studies effectively explained the inhibition mechanism of the most active compounds. It was observed that the presence of groups such as Br, –CH_2_, –NH and C=N^+^ imparted significant inhibitory potential to the screened ligands.

### 2.4. In Vitro α-Glucosidase Inhibition

Benzimidazole serves as a privileged nucleus for antidiabetic drugs, and it is worth mentioning that the majority of small-molecule drugs approved by the US FDA (United States Food and Drug Administration) are derived from this heterocycle [41]. Moreover, C^2^-substituted benzimidazolium salts possess significant α-glucosidase inhibitory profiles in comparison with C^2^-unsubstituted benzimidazole derivatives [29,42]. On the other hand, the pharmacological potential of morpholine is also evident from a number of drugs available commercially such as Gefitinib, Aprepitant and Linezolid [43]. In this context, novel benzimidazolium salts bearing *N*-methylmorpholine at C^2^ were synthesized and screened for their in vitro α-glucosidase inhibitory activity. The calculated IC_50_ values of the newly synthesized salts are shown in Table 2. Particularly, the compound 1-benzyl-3-(2-((4-bromophenyl)amino)-2-oxoethyl)-2-(morpholinomethyl)-1H-benzo[d]imidazol-3-ium chloride **5d** showed the best α-glucosidase inhibitory potential with an IC_50_ value of 15 ± 0.030 µM, which is approximately 4-fold higher than the standard drug (acarbose, IC_50_ = 58.8 ± 0.015 µM). The compound 1-benzyl-3-(2-((3-nitrophenyl)amino)-2-oxoethyl)-2-(morpholinomethyl)-1*H*-benzo[*d*]imidazol-3-ium chloride **5f** was found to have the second most active scaffold with an IC_50_ value of 19 ± 0.060 µM. In addition, benzimidazolium salts **5h**, **5g** and **5k** displayed remarkable α-glucosidase inhibitions with IC_50_ values of 21 ± 0.076, 25 ± 0.106 and 25 ± 0.035 µM, respectively.

It is a well-established fact that the nature as well as the position of substituents on phenyl rings have vital roles in determining the effectiveness of compounds such as α-glucosidase inhibitors [12]. Herein, the structure–activity relationship was found to be in good agreement with the molecular docking results. Benzimidazolium salt **5d,** having a bromo group at the para position, showed the best inhibitory activity, while the rest of the halo-substituted derivatives such as **5a**, **5b** and **5c** exhibited variable (moderate to low) α-glucosidase inhibitory activity. For compounds **5e**, **5f** and **5g** with electron-withdrawing nitro substituents at the ortho, meta and para positions, respectively, a considerable increase in α-glucosidase inhibitory activity was observed. Thus, these three analogs were categorized as moderate to good α-glucosidase inhibitors. Among the compounds **5h**–**5l** possessing electron-donating substituents (methyl or methoxy substituents), only the ortho-substituted derivatives **5h** and **5k** showed considerable α-glucosidase inhibitory profiles.

## 3. Materials and Methods

### 3.1. General

The ^1^H NMR and ^13^C NMR spectra were recorded on a Bruker DRX-600 Topspin in DMSO-*d*6 using TMS as the internal standard. Chemical shift (δ) and coupling constant (*J*) values were documented in parts per million (ppm) and Hertz (Hz), respectively. Melting points were determined using the open capillary tube method. Merck silica-gel 60F-254 plates were used to perform TLC (visualized under UV lamp), while column chromatography was performed with Scharlau Silica Gel 60 N.

### 3.2. Synthesis of 4-((1H-benzimidazol-2-yl)methyl)morpholine (***2***)

2-Chloromethyl-1*H*-benzimidazole **1** (8.4 g, 0.050 mol) was dissolved in 60 mL of acetonitrile followed by the addition of potassium carbonate (7 g, 0.05 mol). After that, morpholine (4.35 mL, 0.050 mol) was added drop-wise with continuous stirring, and the reaction mixture was refluxed overnight. The progress of the reaction was constantly monitored by TLC (chloroform: methanol, 4:1). After the completion of the reaction, the mixture was allowed to cool, and solvent was removed using a rotary evaporator. Finally, the obtained crude solid was purified via column chromatography and recrystallized with ethanol. Yield: 60%; ^1^H NMR (500 MHz, DMSO) δ: 2.51 (s, 4H, morpholine), 3.60 (s, 4H, morpholine), 4.40 (s, 2H, CH_2_-morpholine), 7.23–7.26 (m, 2H, Ar-H), 7.49–7.51 (m, 2H, Ar-H), 9.78 (s, 1H, NH). ^13^C NMR (150 MHz, DMSO) δ 49.20, 51.26, 61.81, 65.12, 70.25, 113.29, 124.32 (2C), 126.92, 127.97, 128.26, 134.88, MS (ESI+): *m*/*z* calcd for C_12_H_15_N_3_O [M + H]: 218; found 218.

### 3.3. Synthesis of 4-((1-benzyl-1H-benzimidazol-2-yl)methyl)morpholine (***3***)

4-((1*H*-Benzimidazol-2-yl)methyl)morpholine **2** (3 g, 0.01 mol) was dissolved in acetonitrile (50 mL), and potassium carbonate (3.58 g, 0.02 mol) was added into the solution. Afterwards, benzyl chloride (0.01 mol) was added drop-wise to the reaction mixture and refluxed for 16 h. Then, the reaction mixture was cooled, and acetonitrile (solvent) was evaporated. Residue solid was subjected to column chromatography, and the pure product was recrystallized with ethanol. Yield: 65%; ^1^H NMR (500 MHz, DMSO) δ: 2.32 (s, 4H, morpholine), 3.60 (s, 4H, morpholine), 4.33 (s, 2H, CH_2_-morpholine), 5.56 (s, 2H, CH_2_-Ph), 7.19–7.24 (m, 3H, Ar-H), 7.31–7.35 (m, 2H, Ar-H), 7.50–7.54 (m, 4H, Ar-H). ^13^C NMR (150 MHz, DMSO) δ: 48.00, 49.51, 52.57, 53.78, 58.71, 60.50, 114.13, 114.27, 122.34 (2C), 123.58 (3C), 125.43 (3C), 131.28 (2C), 138.60, MS (ESI+): *m*/*z* calcd for C_19_H_21_N_3_O [M + H]: 308; found 308.

### 3.4. General Procedure for the Preparation of Benzimidazolium Salts (***5a**–**m***)

The synthesized 4-((1-benzyl-1*H*-benzimidazol-2-yl)methyl)morpholine **3** was dissolved in 30 mL of acetonitrile with stirring at 50–60 °C. 2-Chloro-*N*-arylacetamides **4a**–**m** (1 mmol) were then added, and the reaction mixtures were refluxed overnight. The completion of the reaction was observed by TLC (chloroform:methanol, 4:1 ratio). Finally, the flask contents were cooled in an ice bath to obtain the precipitates of crude solid salts, which were purified via column chromatography.

*1-Benzyl-3-(2-((2-chlorophenyl)amino)-2-oxoethyl)-2-(morpholinomethyl)-1H-benzo[d]imidazol-3-ium chloride* (**5a**): Yellowish powder, yield: 60%; ^1^H NMR (500 MHz, DMSO) δ: 2.42 (s, 4H, morpholine), 3.35 (s, 4H, morpholine), 4.31 (s, 2H, CH_2_-morpholine), 5.78 (s, 2H, CH_2_-Ph), 5.99 (s, 2H, CH_2_-CO), 7.22 (t, *J* = 7.7 Hz, 1H, Ar-H), 7.31–7.36 (m, 4H, Ar-H), 7.37–7.42 (m, 2H, Ar-H), 7.55 (d, *J* = 6.7 Hz, 1H, Ar-H), 7.67–7.72 (m, 2H, Ar-H), 7.87 (d, *J* = 8.2 Hz, 1H, Ar-H), 7.98 (d, *J* = 8.0 Hz, 1H, Ar-H), 8.12 (d, *J* = 8.2 Hz, 1H, Ar-H), 10.36 (s, 1H, NH). ^13^C NMR (125 MHz, DMSO) δ 49.10, 50.56, 52.94, 60.71, 66.11, 70.25, 72.71, 114.14, 114.19, 125.32, 125.92, 126.97, 127.36, 127.54 (2C), 128.13, 128.86, 129.25, 129.42 (2C), 130.26, 131.39, 132.79, 134.78, 134.88, 150.99, 164.92 (C=O). MS (ESI+): *m*/*z* calcd for C_27_H_28_ClN_4_O_2_^+^ [M − Cl]^+^: 475, found 475.

*1-Benzyl-3-(2-((4-chlorophenyl)amino)-2-oxoethyl)-2-(morpholinomethyl)-1H-benzo[d]imidazol-3-ium chloride* (**5b**): Yellowish powder, yield: 65%; ^1^H NMR (500 MHz, DMSO) δ: 2.39 (s, 4H, morpholine), 3.25 (s, 4H, morpholine), 4.34 (s, 2H, CH_2_-morpholine), 5.75 (s, 2H, CH_2_-Ph), 5.98 (s, 2H, CH_2_-CO), 7.22–7.28 (m, 5H, Ar-H), 7.40–7.44 (m, 2H, Ar-H), 7.58 (d, *J* = 6.7 Hz, 1H, Ar-H), 7.69–7.73 (m, 2H, Ar-H), 7.80 (d, *J* = 8.2 Hz, 1H, Ar-H), 7.92 (d, *J* = 8.0 Hz, 1H, Ar-H), 8.13 (d, *J* = 8.2 Hz, 1H, Ar-H), 10.42 (s, 1H, NH). ^13^C NMR (125 MHz, DMSO) δ: 48.15, 51.66, 523.12, 61.41, 66.41, 70.45, 72.91, 11.64, 114.89, 125.12, 125.52, 125.81, 126.0, 126.14 (2C), 127.13, 127.76, 129.15, 129.55 (2C), 130.36, 131.01, 131.9, 134.68, 134.71, 151.01, 164.12 (C=O). MS (ESI+): *m*/*z* calcd for C_27_H_28_ClN_4_O_2_^+^ [M − Cl]^+^: 475, found 475.

*1-Benzyl-3-(2-((2-bromophenyl)amino)-2-oxoethyl)-2-(morpholinomethyl)-1H-benzo[d]imidazol-3-ium chloride* (**5c**): Yellowish powder, yield: 60%; ^1^H NMR (500 MHz, DMSO) δ: 2.38 (s, 4H, morpholine), 3.24 (s, 4H, morpholine), 4.33 (s, 2H, CH_2_-morpholine), 5.67 (s, 2H, CH_2_-Ph), 5.99 (s, 2H, CH_2_-CO), 7.24–7.29 (m, 3H, Ar-H), 7.38–7.44 (m, 3H, Ar-H), 7.54–7.64 (m, 5H, Ar-H), 8.0 (d, *J* = 7.7 Hz, 1H, Ar-H), 8.15 (d, *J* = 7.6 Hz, 1H, Ar-H), 11.30 (s, 1H, NH)**.** ^13^C NMR (125 MHz, DMSO) δ: 49.2, 49.43, 51.55, 52.71, 61.75, 66.91, 70.24, 72.70, 114.09, 114.25, 115.72, 121.34 (2C), 127.58 (3C), 129.43 (3C), 130.28, 131.28 (2C), 133.21, 133.55, 137.50, 151.90, 165.60 (C=O). MS (ESI+): *m*/*z* calcd for C_27_H_28_BrN_4_O_2_^+^ [M − Cl]^+^: 519, found 519.

*1-Benzyl-3-(2-((4-bromophenyl)amino)-2-oxoethyl)-2-(morpholinomethyl)-1H-benzo[d]imidazol-3-ium chloride* (**5d**): Yellowish powder, yield: 62 %; ^1^H NMR (500 MHz, DMSO) δ: 2.37 (s, 4H, morpholine), 3.25 (s, 4H, morpholine), 4.31 (s, 2H, CH_2_-morpholine), 5.66 (s, 2H, CH_2_-Ph), 5.98 (s, 2H, CH_2_-CO), 7.29–7.34 (m, 3H, Ar-H), 7.36–7.42 (m, 3H, Ar-H), 7.52–7.55 (m, 2H, Ar-H), 7.64–7.71 (m, 3H, Ar-H), 7.99 (d, *J* = 7.7 Hz, 1H, Ar-H), 8.15 (d, *J* = 7.6 Hz, 1H, Ar-H), 11.36 (s, 1H, NH)**.** ^13^C NMR (125 MHz, DMSO) δ: 49.00, 49.41, 50.57, 52.78, 60.71, 65.90, 70.24, 72.70, 114.09, 114.25, 115.72, 121.34 (2C), 127.58 (3C), 129.43 (3C), 131.28, 132.28 (2C), 133.02, 134.85, 138.60, 150.94, 164.55 (C=O). MS (ESI+): *m*/*z* calcd for C_27_H_28_BrN_4_O_2_^+^ [M − Cl]^+^: 519.1390, found 519.1394.

*1-Benzyl-3-(2-((2-nitrophenyl)amino)-2-oxoethyl)-2-(morpholinomethyl)-1H-benzo[d]imidazol-3-ium chloride* (**5e**): Yellowish powder, yield: 55%; ^1^H NMR (500 MHz, DMSO) δ: 2.40 (s, 4H, morpholine), 3.32 (s, 4H, morpholine), 4.30 (s, 2H, CH_2_-morpholine), 5.78 (s, 2H, CH_2_-Ph), 5.98 (s, 2H, CH_2_-CO), 7.30 (d, *J* = 6.8 Hz, 3H, Ar-H), 7.34- 7.44 (m, 5H, Ar-H), 7.68–7.76 (m, 2H, Ar-H), 7.77–7.78 (m, 1H, Ar-H), 7.99 (d, *J* = 9.0 Hz, 1H, Ar-H), 8.03 (d, *J* = 8.2 Hz, 1H, Ar-H), 11.36 (s, 1H, NH). ^13^C NMR (125 MHz, DMSO) δ: 49.10, 50.53, 52.89, 60.71, 66.09, 70.19, 70.25, 72.71, 114.04, 114.16, 125.46, 125.66, 127.48, 127.51 (2C), 127.54, 128.86, 129.42 (2C), 131.42, 132.63, 134.73, 134.85, 142.76, 151.02, 164.96 (C=O). MS (ESI+): *m*/*z* calcd for C_27_H_28_N_5_O_4_^+^ [M − Cl]^+^: 486; found 486.

*1-Benzyl-3-(2-((3-nitrophenyl)amino)-2-oxoethyl)-2-(morpholinomethyl)-1H-benzo[d]imidazol-3-ium chloride* (**5f**): Yellowish powder, yield: 58%; ^1^H NMR (500 MHz, DMSO) δ: 2.39 (s, 4H, morpholine), 3.25 (s, 4H, morpholine), 4.34 (s, 2H, CH_2_-morpholine), 5.75 (s, 2H, CH_2_-Ph), 5.99 (s, 2H, CH_2_-CO), 7.33–7.42 (m, 5H, Ar-H), 7.66–7.73 (m, 3H, Ar-H), 7.95–8.50 (m, 3H, Ar-H), 8.18 (d, *J* = 7.5 Hz, 1H, Ar-H), 8.72 (t, *J* = 2.2 Hz, 1H, Ar-H), 11.98 (s, 1H, NH). ^13^C NMR (125 MHz, DMSO) δ 49.02, 49.43, 50.56, 52.77, 60.71, 62.37, 65.92, 66.49, 70.24, 72.70, 113.48, 114.12, 114.26, 118.70, 125.47 (2C), 127.60, 129.44 (2C), 130.98, 131.29, 133.02, 134.84, 140.37, 148.51, 150.98, 165.32 (C=O). MS (ESI+): *m*/*z* calcd for C_27_H_28_N_5_O_4_^+^ [M − Cl]^+^: 486, found 486.

*1-Benzyl-3-(2-((4-nitrophenyl)amino)-2-oxoethyl)-2-(morpholinomethyl)-1H-benzo[d]imidazol-3-ium chloride* (**5g**): Yellowish powder, yield: 65%; ^1^H NMR (500 MHz, DMSO) δ: 2.38 (s, 4H, morpholine), 3.24 (s, 4H, morpholine), 4.33 (s, 2H, CH_2_-morpholine), 5.75 (s, 2H, CH_2_-Ph), 5.99 (s, 2H, CH_2_-CO), 7.33–7.42 (m, 5H, Ar-H), 7.50–7.60 (m, 1H, Ar-H), 7.66–7.73 (m, 1H, Ar-H), 7.94 (d, *J* = 9.3 Hz, 2H, Ar-H), 8.01 (d, *J* = 7.6 Hz, 1H, Ar-H), 8.17 (d, *J* = 8.4 Hz, 1H, Ar-H), 8.25–8.31 (m, 2H, Ar-H), 12.01 (s, 1H, NH). ^13^C NMR (125 MHz, DMSO) δ 49.00, 49.63, 50.57, 52.76, 58.77, 60.71, 65.91, 70.25, 72.71, 114.14, 114.22, 119.30, 120.35, 125.67, 127.30, 127.48 (2C), 127.62, 129.44 (2C), 131.26, 133.04, 134.83, 143.00, 145.36, 150.95, 165.56 (C=O). MS (ESI+): *m*/*z* calcd for C_27_H_28_N_5_O_4_^+^ [M − Cl]^+^: 486.2136, found 486.2139.

*1-Benzyl-3-(2-((2-methylphenyl)amino)-2-oxoethyl)-2-(morpholinomethyl)-1H-benzo[d]imidazol-3-ium chloride* (**5h**): Pale yellow powder, yield: 65%; ^1^H NMR (500 MHz, DMSO) δ: 2.33 (s, 3H, CH_3_), 2.46 (s, 4H, morpholine), 3.37 (s, 4H, morpholine), 4.34 (s, 2H, CH_2_-morpholine), 5.82 (s, 2H, CH_2_-Ph), 5.99 (s, 2H, CH_2_-CO), 7.10 (td, *J* = 7.4, 1.2 Hz, 1H, Ar-H), 7.15–7.19 (m, 1H, Ar-H), 7.24–7.32 (m, 4H, Ar-H), 7.35–7.41 (m, 3H, Ar-H), 7.53 (d, *J* = 7.9 Hz, 1H, Ar-H), 7.69 (dt, *J* = 15.6, 7.3 Hz, 2H, Ar-H), 7.96 (d, *J* = 8.1 Hz, 1H, Ar-H), 8.14 (d, *J* = 8.2 Hz, 1H, Ar-H), 10.38 (s, 1H, NH). ^13^C NMR (125 MHz, DMSO) δ 18.64, 48.94, 49.15, 50.56, 52.98, 60.27, 60.71, 66.16, 70.25, 72.70, 114.11, 114.21, 124.47, 126.53, 127.44, 127.46 (2C), 127.50, 128.77, 129.38 (2C), 131.03, 131.47, 131.62, 134.95, 136.13, 151.06, 164.31 (C=O). MS (ESI+): *m*/*z* calcd for C_28_H_31_N_4_O_2_^+^ [M − Cl]^+^: 455, found 455.

*1-Benzyl-3-(2-((3-methylphenyl)amino)-2-oxoethyl)-2-(morpholinomethyl)-1H-benzo[d]imidazol-3-ium chloride* (**5i**): Yellowish powder, yield: 55%; ^1^H NMR (500 MHz, DMSO) δ: 2.27 (s, 3H, CH_3_), 2.39 (s, 4H, morpholine), 3.28 (s, 4H, morpholine), 4.32 (s, 2H, CH_2_-morpholine), 5.66 (s, 2H, CH_2_-Ph), 5.98 (s, 2H, CH_2_-CO), 6.91 (d, *J* = 7.5 Hz, 1H, Ar-H), 7.21 (t, *J* = 7.9 Hz, 1H, Ar-H), 7.32–7.34 (m, 2H, Ar-H), 7.36–7.42 (m, 3H, Ar-H), 7.42 (d, *J* = 7.6 Hz, 1H, Ar-H), 7.65–7.72 (m, 2H, Ar-H), 7.99 (d, *J* = 8.4 Hz, 1H, Ar-H), 8.15 (d, *J* = 8.5 Hz, 1H, Ar-H), 11.07 (s, 1H, NH). ^13^C NMR (125 MHz, DMSO) δ 21.66 49.01, 49.37, 50.58, 52.82, 65.90, 70.25, 114.06, 114.29, 116.58, 117.58, 119.88, 124.84, 127.26, 127.45, 127.57 (2C), 128.88, 129.26, 129.43 (2C), 131.31, 132.99, 134.89, 138.62, 139.13, 150.95, 164.19 (C=O). MS (ESI+): *m*/*z* calcd for C_28_H_31_N_4_O_2_^+^ [M − Cl]^+^: 455, found 455.

*1-Benzyl-3-(2-((4-methylphenyl)amino)-2-oxoethyl)-2-(morpholinomethyl)-1H-benzo[d]imidazol-3-ium chloride* (**5j**): Yellowish powder, yield: 60%; ^1^H NMR (500 MHz, DMSO) δ: 2.25 (s,3H, CH_3_), 2.34 (d, *J* = 8.2 Hz, 4H, morpholine), 3.32 (s, 4H, morpholine), 3.95 (s, 2H, CH_2_-morpholine), 5.69 (s, 2H, CH_2_-Ph), 5.98 (s, 2H, CH_2_-CO), 7.12 (d, *J* = 8.4 Hz, 1H, Ar-H, 7.19 (d, *J* = 8.3 Hz, 2H, Ar-H), 7.31–7.42 (m, 7H, Ar-H), 7.51–7.58 (m, 2H, Ar-H), 7.64 (d, *J* = 8.9 Hz, 1H, Ar-H), 11.28 (s, 1H, NH). MS (ESI+): *m*/*z* calcd for C_28_H_31_N_4_O_2_^+^ [M − Cl]^+^: 455, found 455.

*1-Benzyl-3-(2-((2-methoxyphenyl)amino)-2-oxoethyl)-2-(morpholinomethyl)-1H-benzo[d]imidazol-3-ium chloride* (**5k**): White crystalline solid, yield: 60%; ^1^H NMR (500 MHz, DMSO) δ: 2.40 (s, 4H, morpholine), 3.51 (s, 4H, morpholine), 3.91 (s, 3H, OCH_3_), 4.38 (s, 2H, CH_2_-morpholine), 5.74 (s, 2H, CH_2_-Ph), 5.98 (s, 2H, CH_2_-CO), 6.95–6.88 (m, 3H, Ar-H), 7.07 (dd, *J* = 8.2, 1.4 Hz, 3H, Ar-H), 7.10–7.14 (m, 4H, Ar-H), 7.32–7.42 (m, 3H, Ar-H), 10.00 (s, 1H, NH). ^13^C NMR (125 MHz, DMSO) δ 41.95, 43.84 (2C), 49.09, 49.33, 50.55, 52.89 (3C), 56.23, 65.94, 70.27, 111.73, 120.80 (2C), 121.63 (2C), 122.05 (2C), 125.27, 125.50, 126.99, 127.28, 127.56, 128.85, 129.41, 131.37, 132.91, 134.89, 150.07, 165.22 (C=O). MS (ESI+): *m*/*z* calcd for C_28_H_31_N_4_O_3_^+^ [M − Cl]^+^: 471, found 471. 

*1-Benzyl-3-(2-((4-methoxyphenyl)amino)-2-oxoethyl)-2-(morpholinomethyl)-1H-benzo[d]imidazol-3-ium chloride* (**5l**): White crystalline solid, yield: 65%; ^1^H NMR (500 MHz, DMSO) δ: 2.39 (s, 4H, morpholine), 3.29 (s, 4H, morpholine), 3.73 (s, 3H, OCH_3_), 4.31 (s, 2H, CH_2_-morpholine), 5.59 (s, 2H, CH_2_-Ph), 5.98 (s, 2H, CH_2_-CO), 6.91 (d, *J* = 8.6 Hz, 2H, Ar-H), 7.32–7.42 (m, 6H, Ar-H), 7.56 (d, *J* = 9.1 Hz, 1H, Ar-H), 7.66–7.72 (m, 2H, Ar-H), 7.99 (d, *J* = 7.4 Hz, 1H, Ar-H), 8.13 (d, *J* = 8.5 Hz, 1H, Ar-H), 10.82 (s, 1H, NH). ^13^C NMR (125 MHz, DMSO) δ 49.01, 49.26, 50.58, 52.85, 55.62 (2C), 65.92, 70.25, 72.71, 114.07, 114.56 (2C), 120.82 (2C), 127.27, 127.45 (2C), 127.56, 128.88, 129.43 (2C), 131.32, 132.28, 132.99, 134.89, 150.93, 155.88, 166.79 (C=O). MS (ESI+): *m*/*z* calcd for C_28_H_31_N_4_O_3_^+^ [M − Cl]^+^: 471, found 471.

*1-Benzyl-2-(morpholinomethyl)-3-(2-oxo-2-(phenylamino)ethyl)-1H-benzo[d]imidazol-3-ium chloride* (**5m**): White crystalline solid, yield: 60%; ^1^H NMR (500 MHz, DMSO) δ: 2.39 (s, 4H, morpholine), 3.28 (s, 4H, morpholine), 4.32 (s, 2H, CH_2_-morpholine), 5.64 (s, 2H, CH_2_-Ph), 5.98 (s, 2H, CH_2_-CO), 7.09 (t, *J* = 6.9 Hz, 1H, Ar-H), 7.32–7.38 (m, 5H, Ar-H), 7.39–7.42 (m, 2H, Ar-H), 7.65–7.72 (m, 4H, Ar-H), 8.00 (d, *J* = 8.5 Hz, 1H, Ar-H), 8.14 (d, *J* = 7.6 Hz, 1H, Ar-H), 11.02 (s, 1H, NH). ^13^C NMR (125 MHz, DMSO) δ 49.01, 49.38, 50.59, 52.83, 60.72, 65.90, 70.25, 72.71, 114.09, 114.26, 119.37, 124.15, 127.27, 127.47, 127.58, 128.89, 129.43, 129.46, 129.55, 131.31, 133.00, 134.88, 139.15, 150.94, 164.23 (C=O). MS (ESI+): *m*/*z* calcd for C_27_H_29_N_4_O_2_^+^ [M + H-Cl]^+^: 441, found 442.

### 3.5. Biological Evaluation

#### 3.5.1. In Silico α-Glucosidase Inhibition Studies

The molecular docking of potent benzimidazolium salts along with the reference drug, acarbose, against the α-glucosidase enzyme was performed via MOE software (Montreal, Canada), Ver.2014.0901 [44,45] according to our reported protocol [37]. The chemical structures of all the compounds were drawn by using ChemDraw Ultra 12.02 and were saved as MDL files (“.sdf”). The NCBI Pubchem database was accessed to download the two-dimensional structure of acarbose in the ‘sdf’ format. The Protein Data Bank (http://www.rcsb.org/pdb; accessed on 2 April 2022) was accessed to retrieve the 3D structure of α-glucosidase (PDB ID: 2QMJ). Acarbose, the standard drug, displayed the binding interaction with Asp203, Asp542, Asp327, His600 and Arg526 (α-glucosidase residues). All the ligands (the synthesized compounds), reference acarbose and the receptor protein were 3D-protonated and energy minimized by using MMFF94X force field. The docking was performed by setting following parameters; rescoring: London dG, placement: triangle matcher, retain: 10, and refinement: forcefield. The leading conformation of the docked ligands was selected on the basis of binding energies, RMSD values and interaction modes with the selected residues.

#### 3.5.2. In Vitro α-Glucosidase Inhibition Studies

α-Glucosidase inhibitions were determined following a modified spectroscopic method [46]. The detailed protocol was already reported [29]. Each experiment was carried out thrice, and percentage inhibitions were determined with the help of the following expression, followed by the determination of IC_50_ values:

% Inhibition = [(A_control_ − A_sample_)/A_control_] × 100

where ‘A’ stands for absorbance.

## 4. Conclusions

In summary, thirteen *N*-methylmorpholine (NMM)-based benzimidazolium salts were synthesized by using a multistep approach starting from 2-chloromethyl- 1*H*-benzimidazole **1**. These synthesized novel compounds were characterized and evaluated for their α-glucosidase inhibitory potential. The outcome of the study, correlated with the docking study, revealed that the compound 1-benzyl-3-(2-((4-bromophenyl)amino)-2-oxoethyl)-2-(morpholinomethyl)-1*H*-benzo[*d*]imidazol-3-ium chloride **5d** with the lowest IC_50_ value of 15 ± 0.030 µM possessed potent inhibitory potential against the α-glucosidase enzyme. Furthermore, compounds **5f**, **5h**, **5g**, **5k** and **5e** also showed moderate to good α-glucosidase inhibitory activity with IC_50_ values of 19 ± 0.060 µM, 21 ± 0.076 µM, 25 ± 0.106 µM, 25 ± 0.035 µM and 30 ± 0.110 µM, respectively.

## Figures and Tables

**Table 1:** The in silico screening data of potent *N*-methylmorpholine-substituted benzimidazolium salts and acarbose.

Compound	5d	5f	5g	5h	5k	Acarbose
Docking Score (Kcal/mol)	−13.15	−13.10	−12.10	−12.75	−12.15	−13.87
Rmsd value (Å)	1.01	1.15	1.45	1.19	1.38	2
Interacting Residues	Asp203, Asp327	Asp203, Asp542	Asp203, Asp542	Asp203	Asp203	His600, Asp542, Arg526, Asp327, Met444, Lys480

**Table 2:** In vitro α-glucosidase inhibition (IC_50_) of synthesized benzimidazolium salts **5a**–**m**.

Compound	R	IC_50_ (µM)	Compound	R	IC_50_ (µM) *
**5a**	2-Cl	50 ± 0.040	**5h**	2-CH_3_	21 ± 0.076
**5b**	4-Cl	35 ± 0.020	**5i**	3-CH_3_	47 ± 0.050
**5c**	2-Br	55 ± 0.140	**5j**	4-CH_3_	88 ± 0.080
**5d**	4-Br	15 ± 0.030	**5k**	2-OCH_3_	25 ± 0.035
**5e**	2-NO_2_	30 ± 0.110	**5l**	4-OCH_3_	65 ± 0.015
**5f**	3-NO_2_	19 ± 0.060	**5m**	H	110 ± 0.113
**5g**	4-NO_2_	25 ± 0.106	**Acarbose**	-	58.8 ± 0.015

* All experiments were conducted in replicate. Means of experiments are presented, *n* = 3 and *p* < 0.05.

## Data Availability

The data presented in this study are available in the Supplementary Materials.

## Supplementary Materials

The following are available online at https://www.mdpi.com/article/10.3390/molecules27186012/s1. The ^1^H-NMR, ^13^C-NMR and MS spectra of the synthesized compounds with potent enzyme inhibition activities.
