# Supplementary material for: Synthesis of Novel N-Methylmorpholine-Substituted Benzimidazolium Salts as Potential α-Glucosidase Inhibitors
Source: Molecules. 2022 Sep 15;27(18):6012. doi: 10.3390/molecules27186012 (PMC9501035; doi:10.3390/molecules27186012)
Supplement: Supplementary file 1 [file molecules-27-06012-s001.zip › molecules-1875702-supplementary.pdf]

Synthesis of Novel *N*-methylmorpholine Substituted Benzimidazolium Salts as Potential *α*-Glucosidase Inhibitors

Imran Ahmad Khan [1], Furqan Ahmad Saddique [1], Sana Aslam [2], Usman Ali Ashfaq [3], Matloob Ahmad [1,*], Sami A. Al-Hussain [4], Magdi E. A. Zaki [4,*]

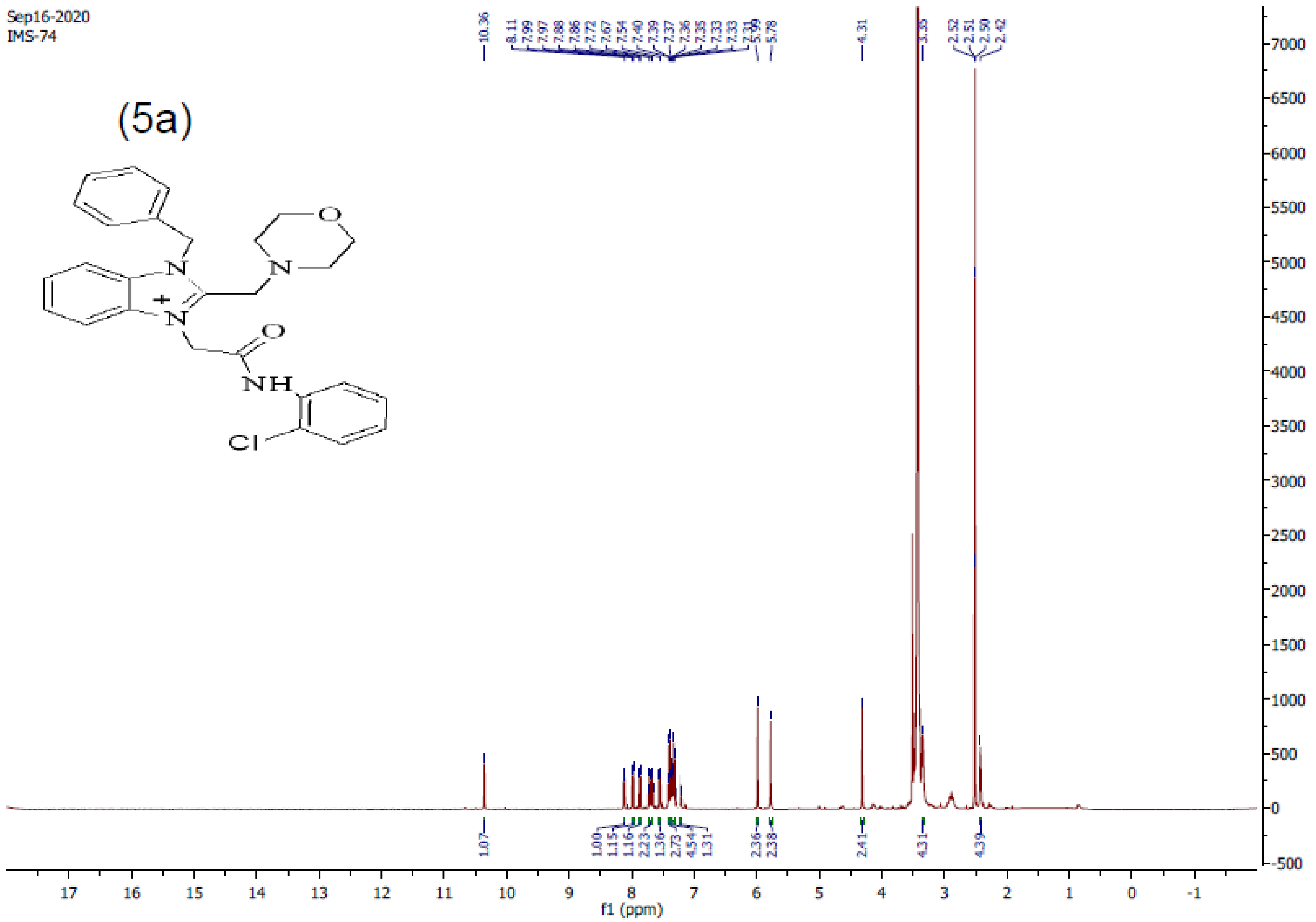

Figure S1. ^{1}H NMR Spectra of compound **5a**

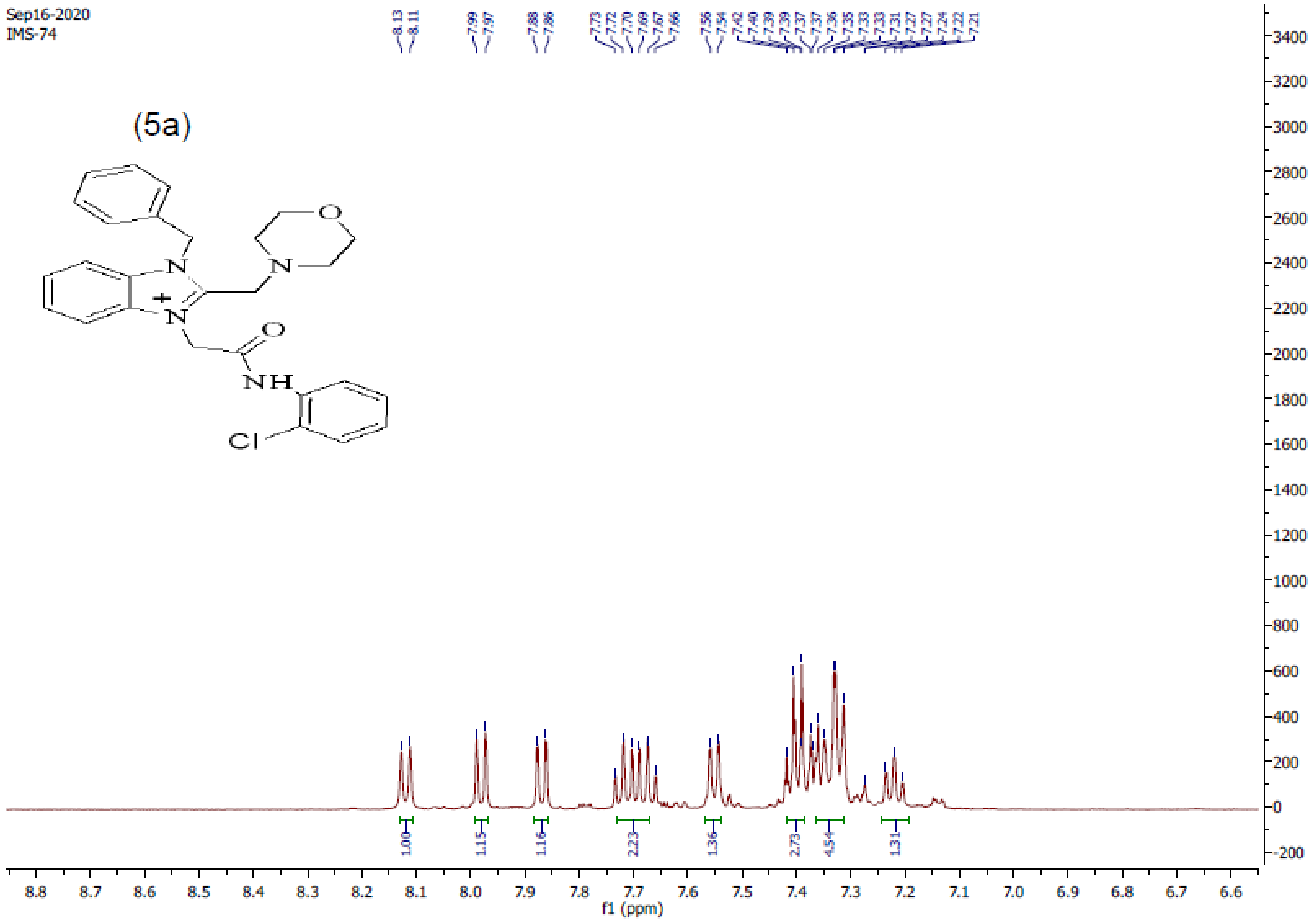

Figure S1a. Expanded version of ^{1}H NMR Spectra of compound **5a**

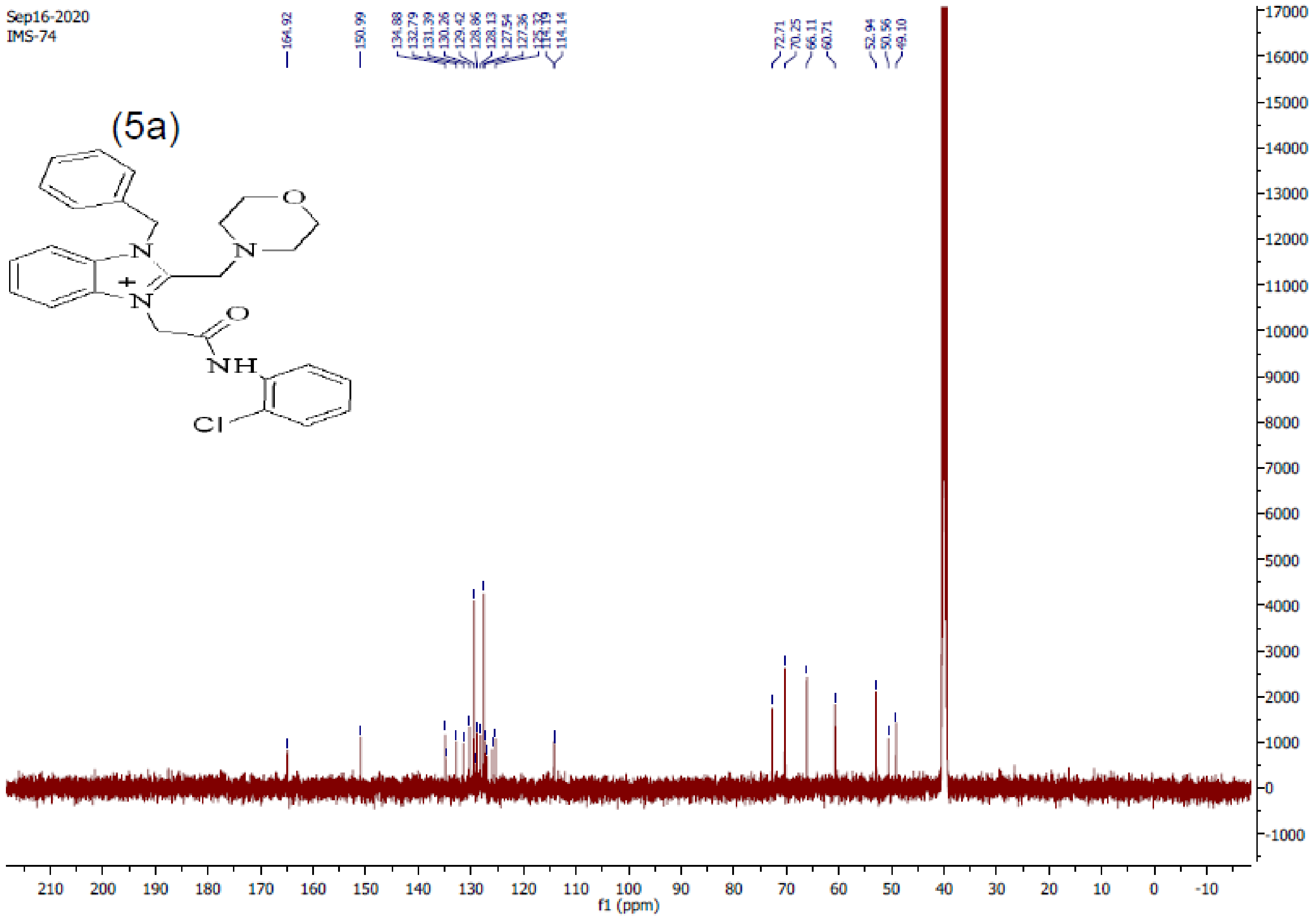

Figure S2. ^{13}C NMR Spectra of compound **5a**

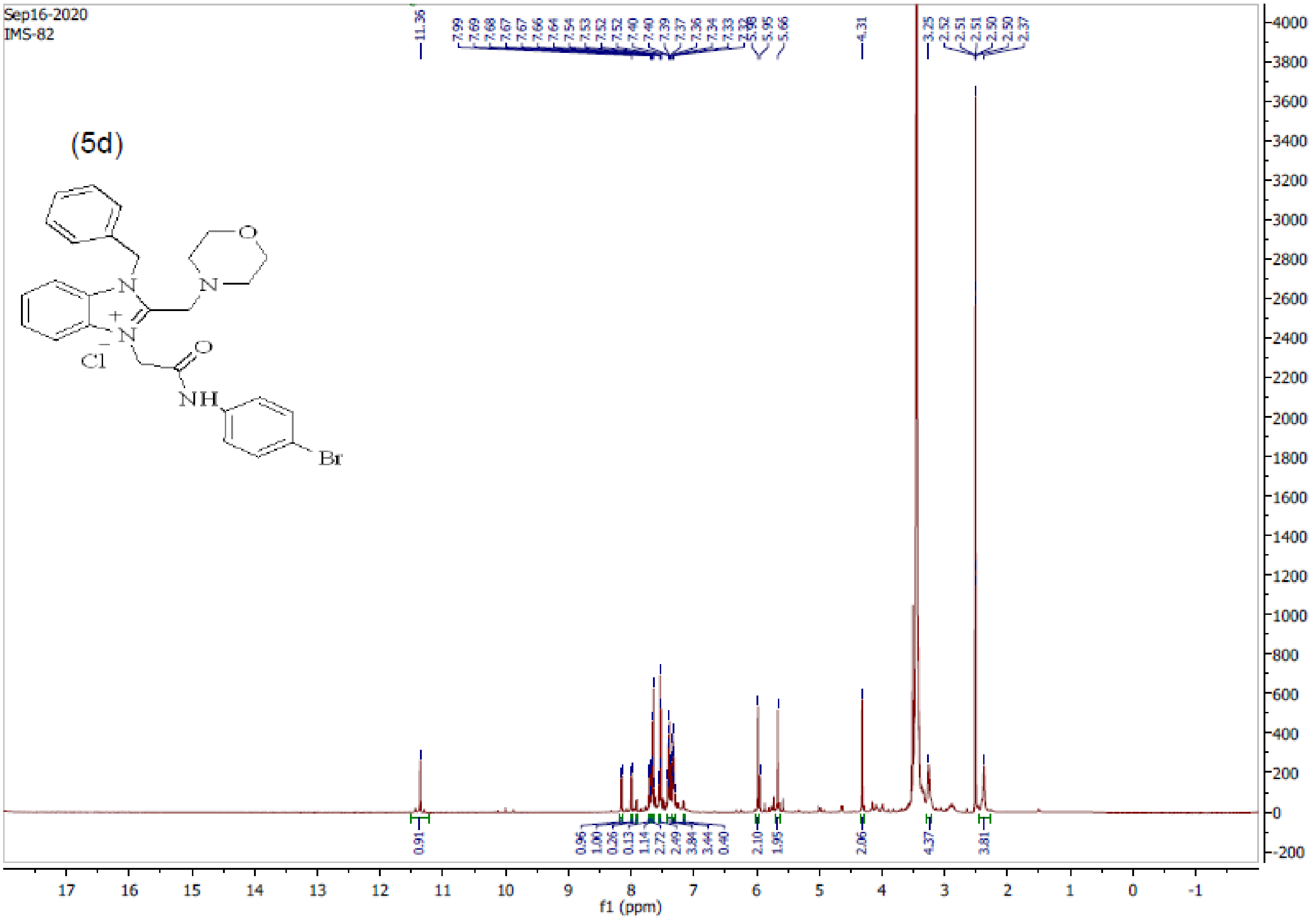

Figure S3. ^{1}H NMR Spectra of compound **5d**

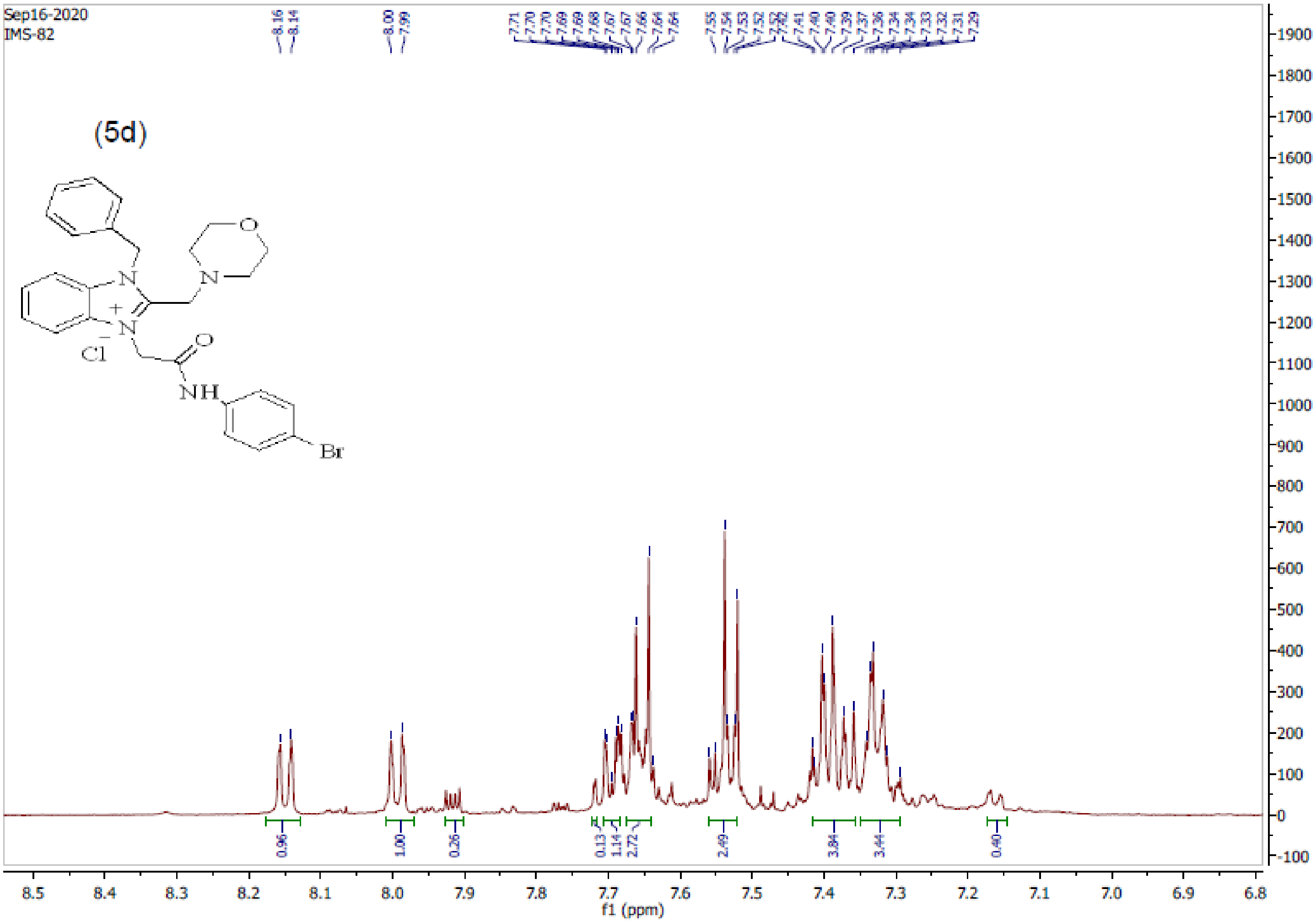

Figure S3a. Expanded version of ^{1}H NMR Spectra of compound **5d**

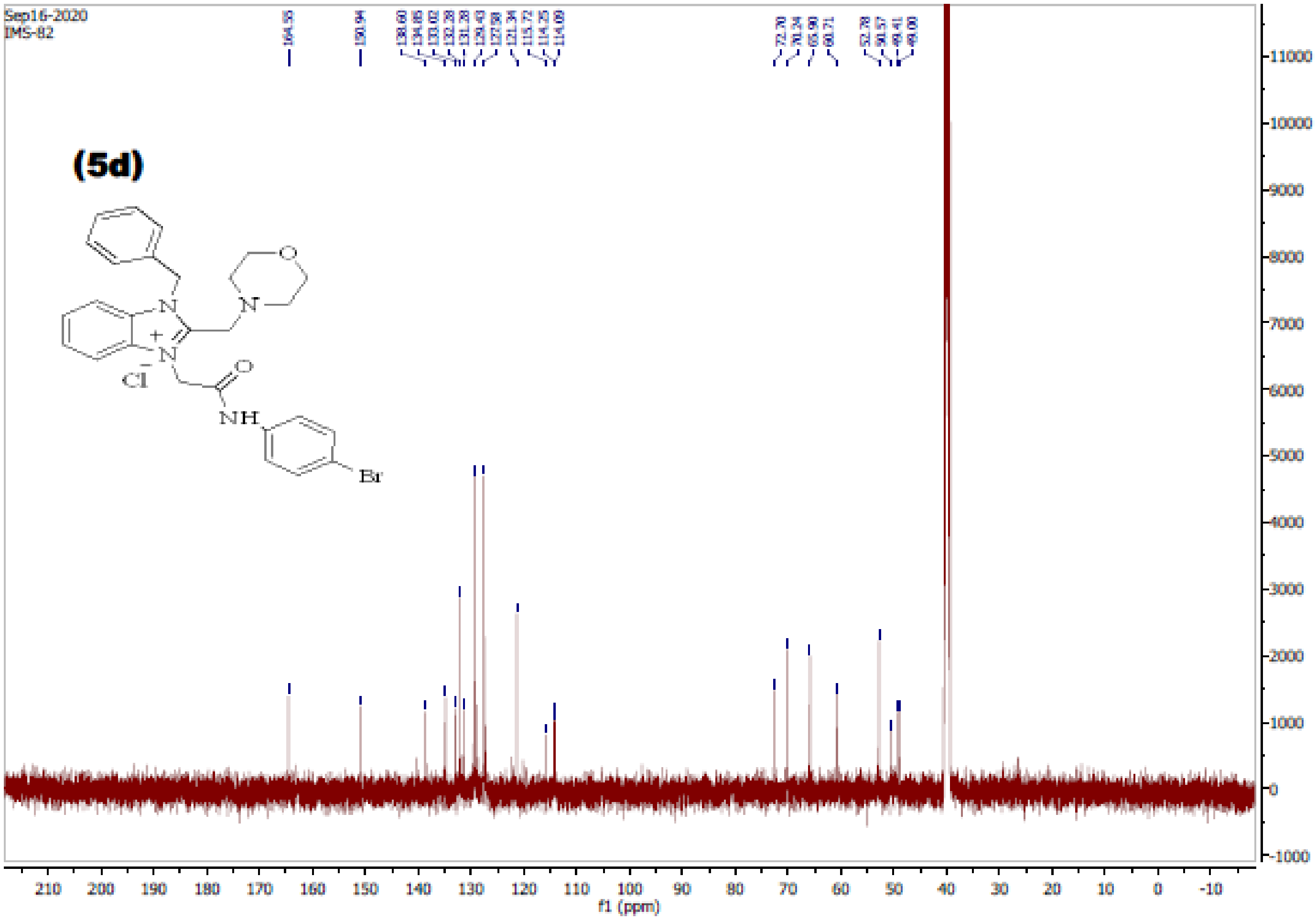

Figure S4. ^{13}C NMR Spectra of compound **5d**

(5d)

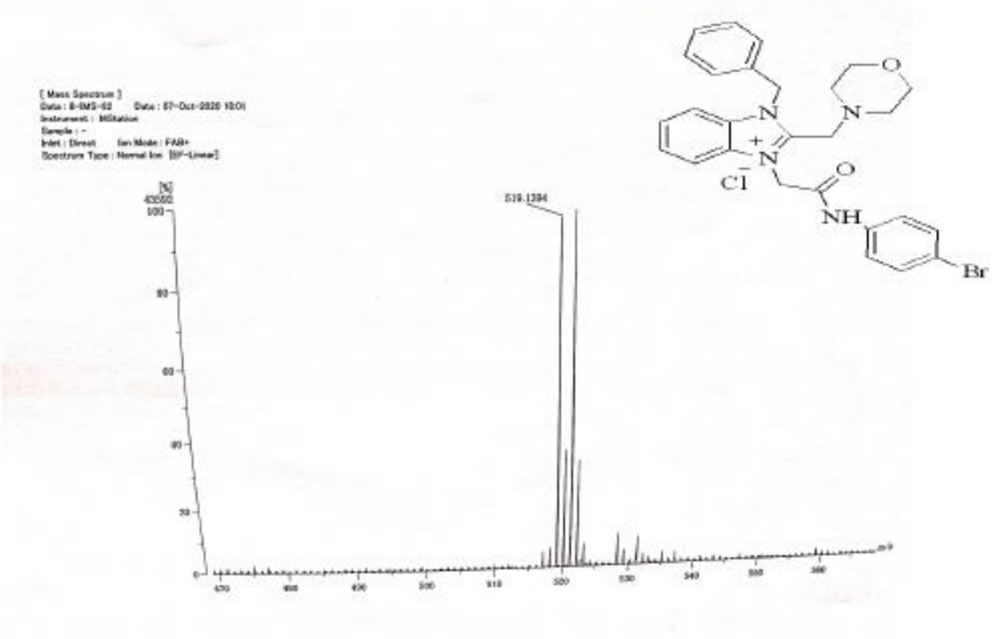

Scanned with CamScanner

Figure S5. MS (ESI+) Spectra of compound **5d**

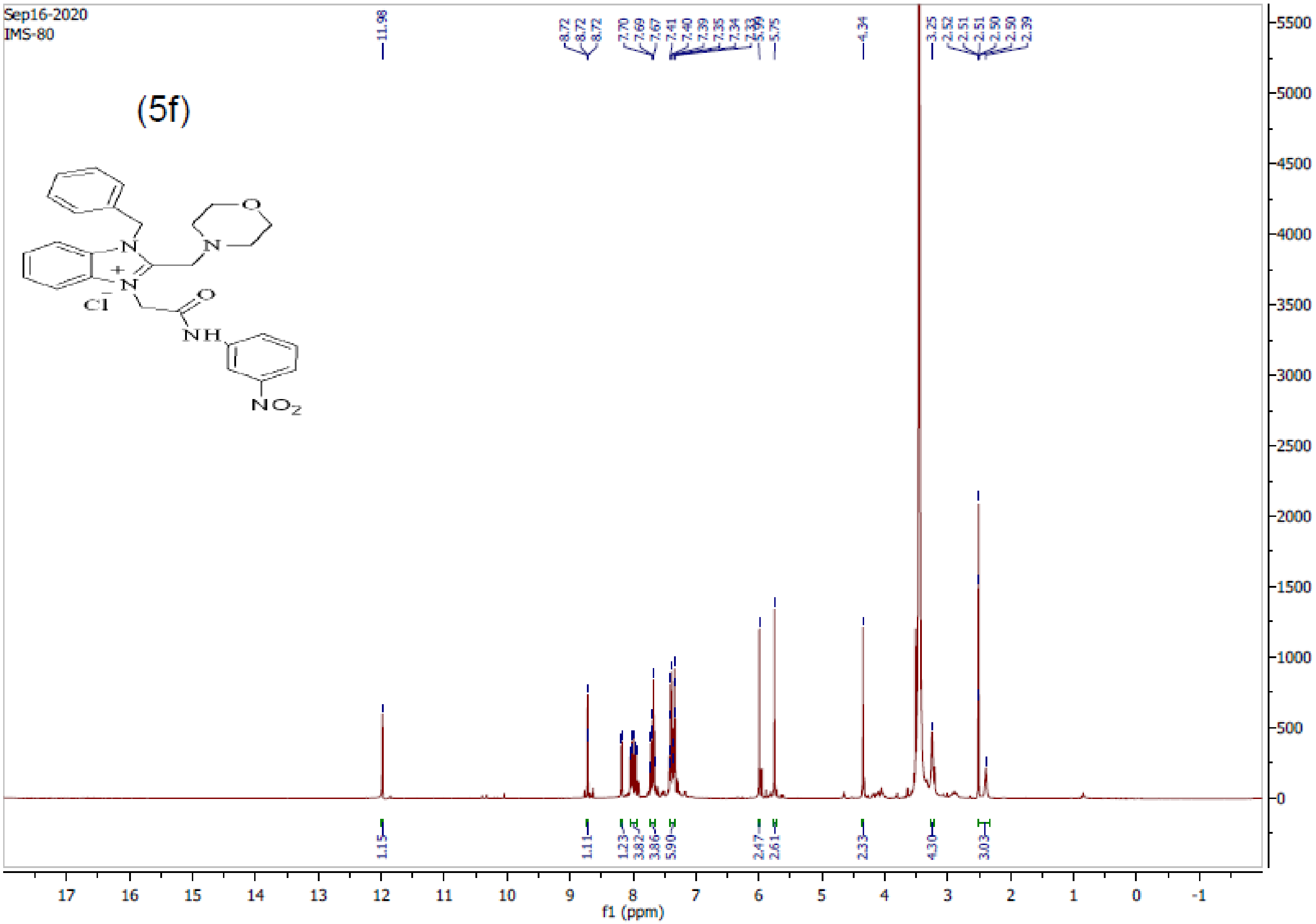

Figure S6. ^{1}H NMR Spectra of compound **5f**

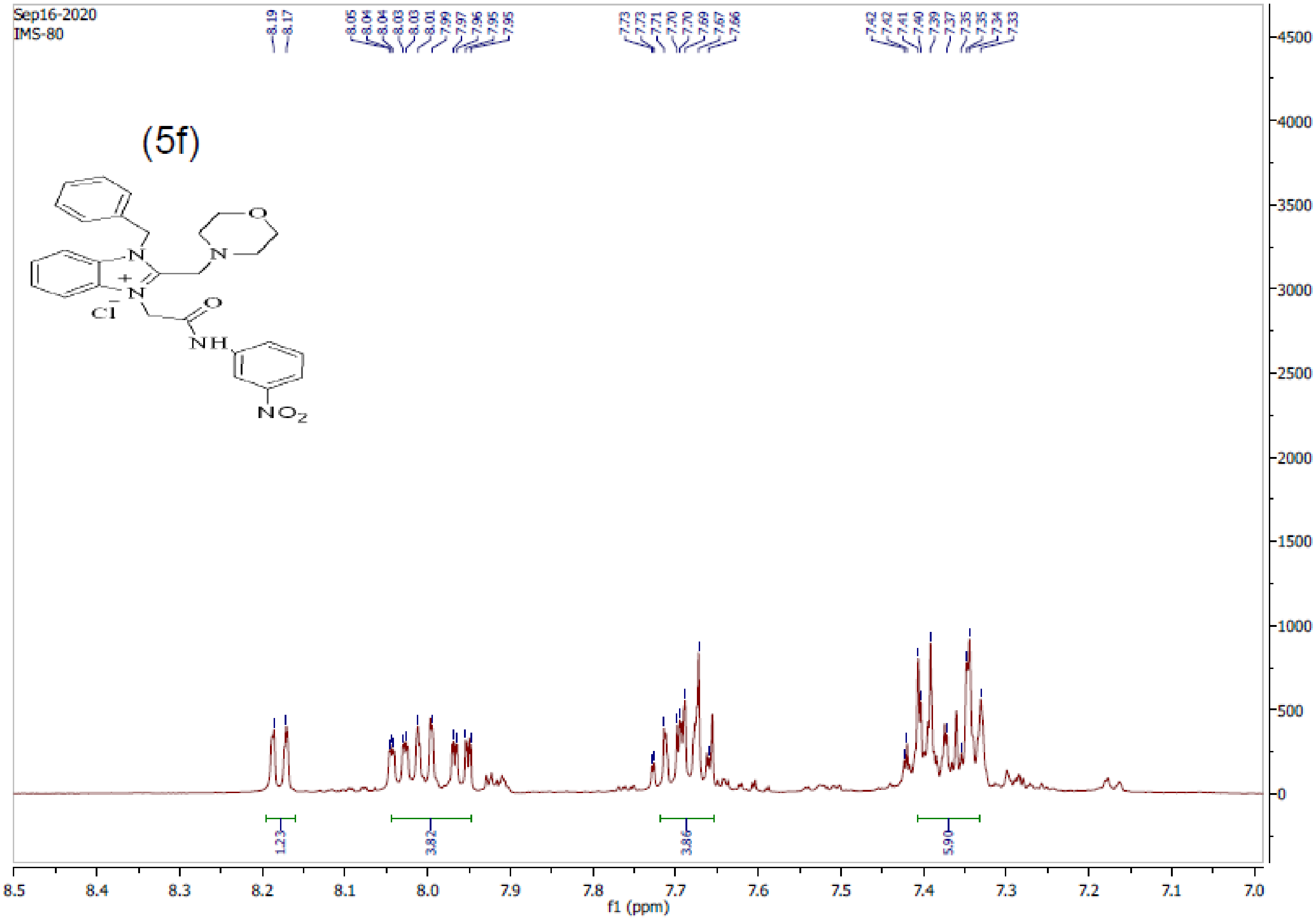

Figure S6a. Expanded version of ^{1}H NMR Spectra of compound **5f**

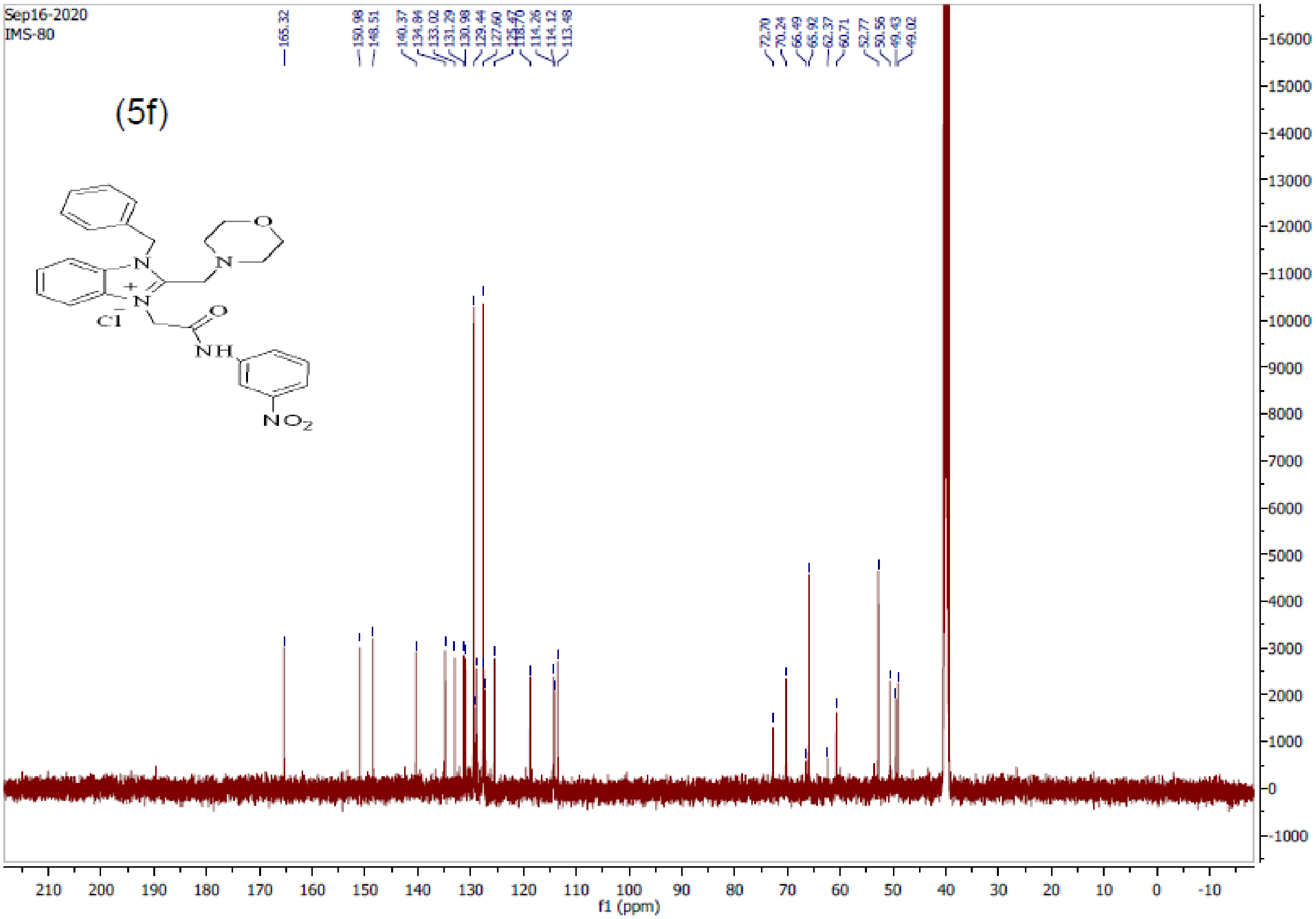

Figure S7. ^{13}C NMR Spectra of compound **5f**

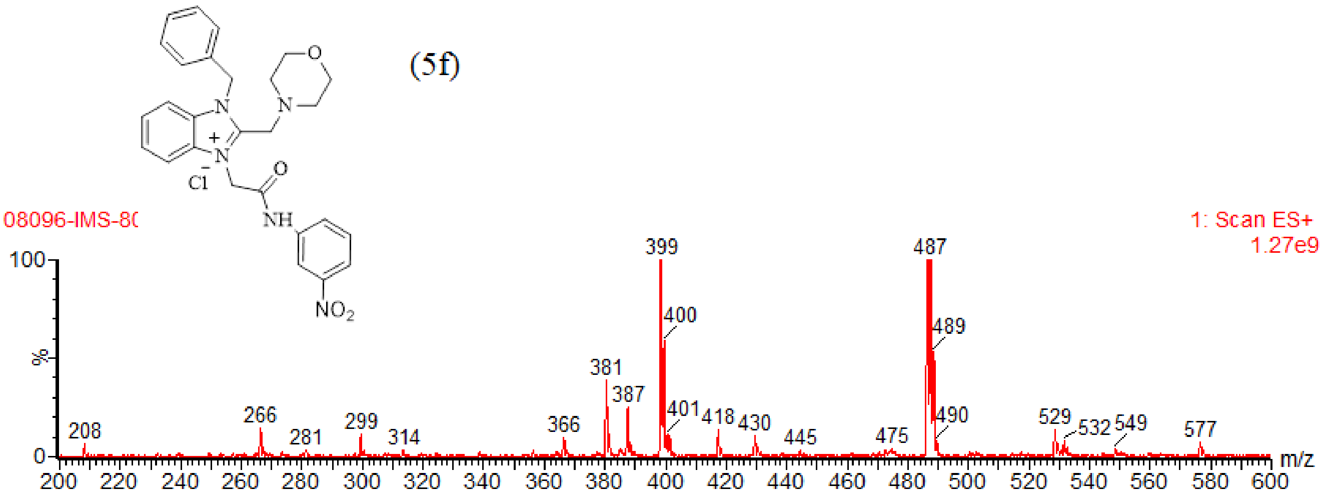

Figure S8. MS (ESI+) Spectra of compound **5f**

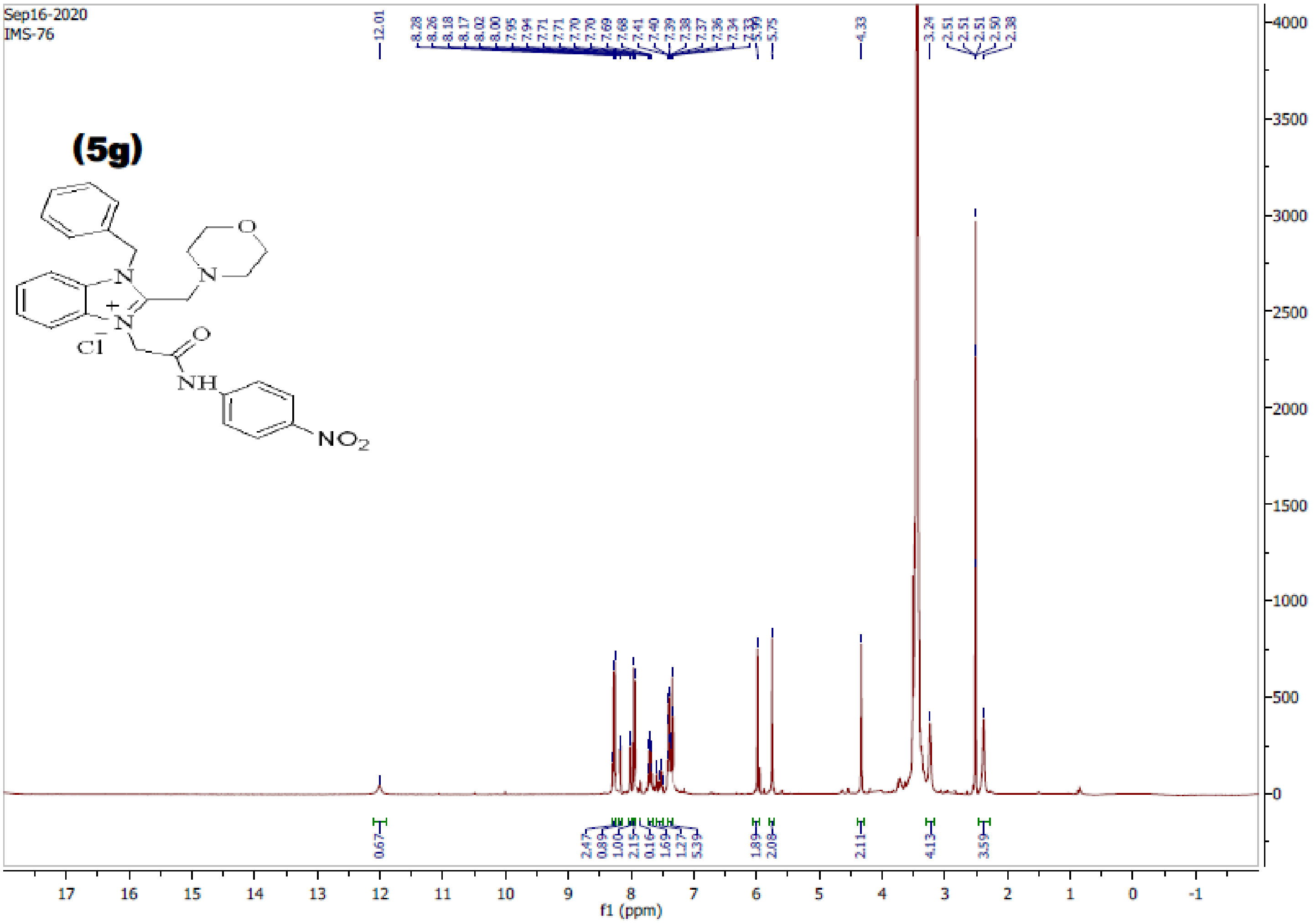

Figure S9. [1]*H* NMR Spectra of compound **5g**

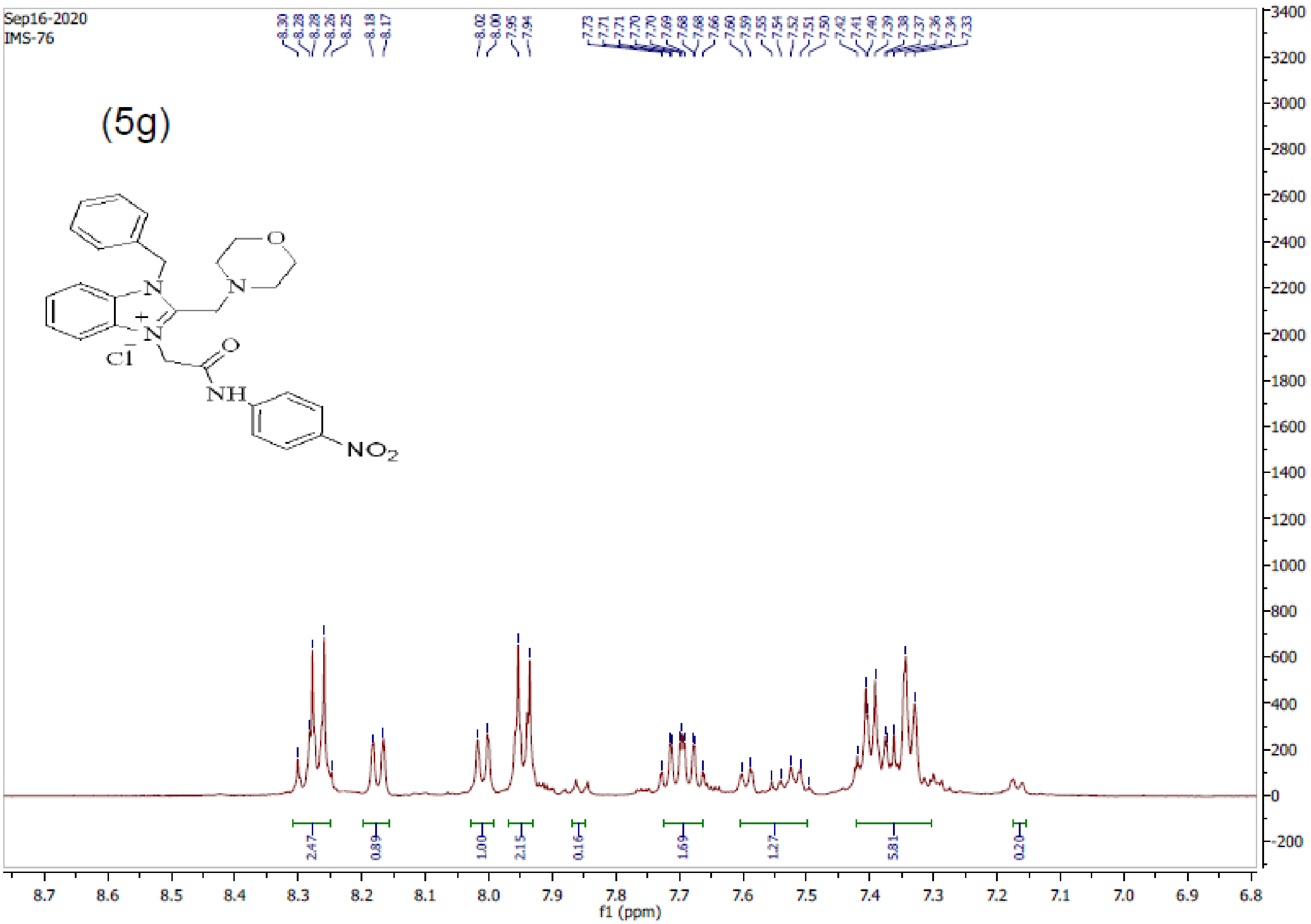

Figure S9a. Expanded version of ^{1}H NMR Spectra of compound **5g**

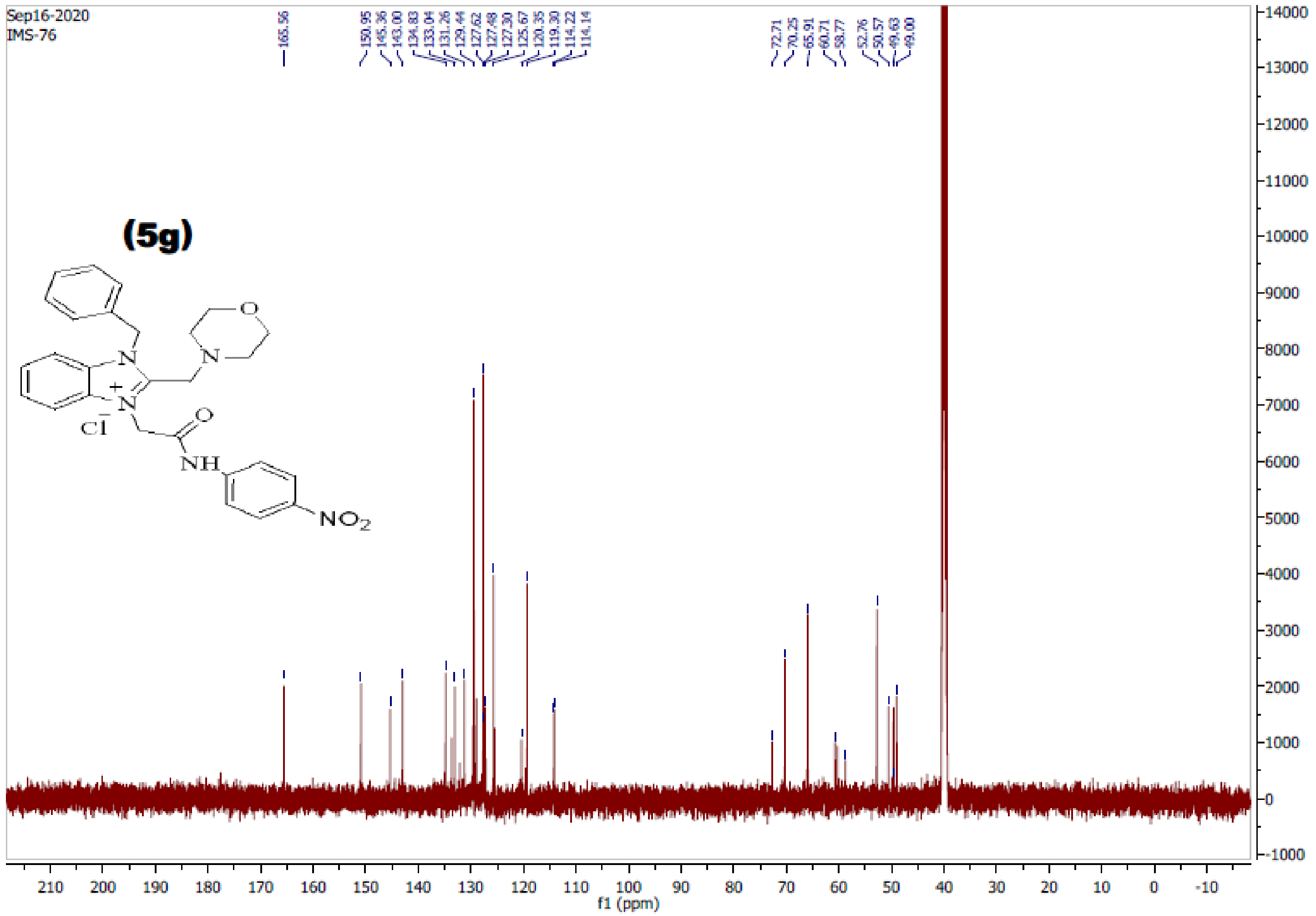

Figure S10. ^{13}C NMR Spectra of compound **5g**

(5g)

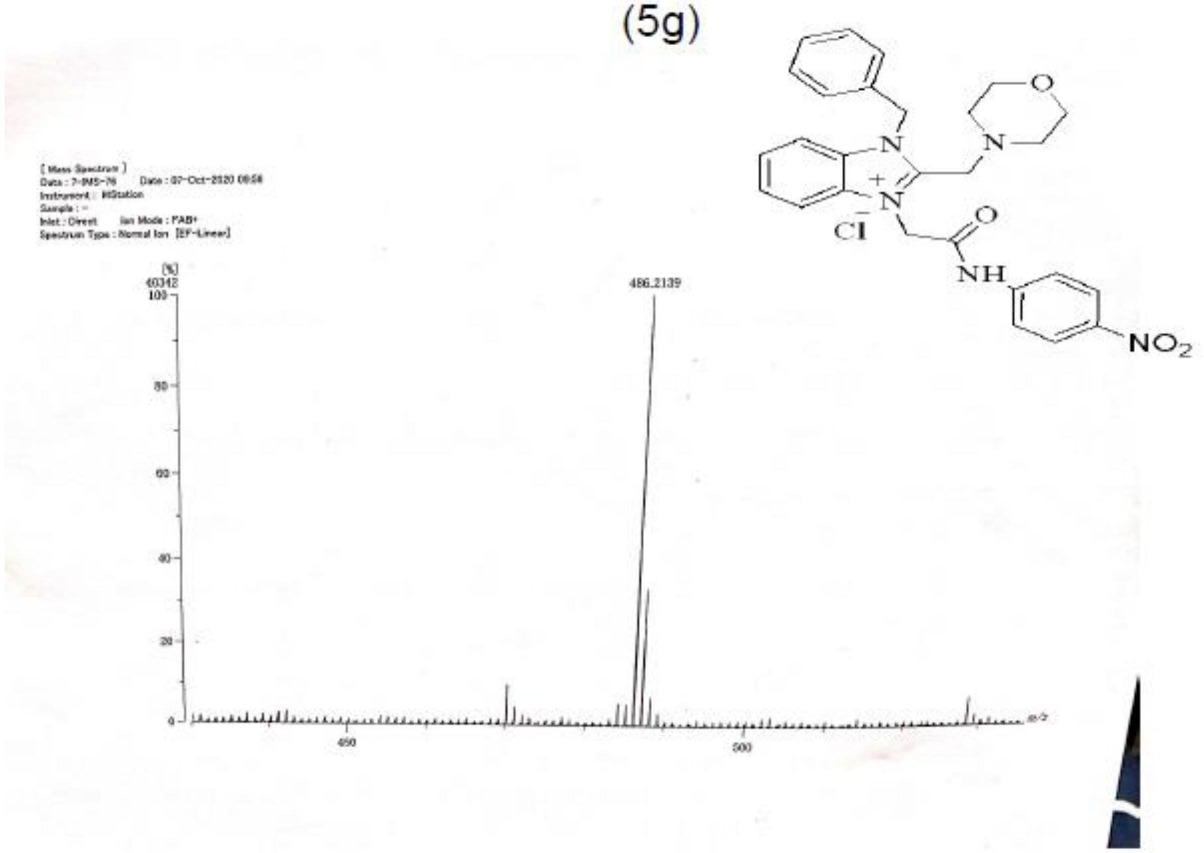

Scanned with CamScanner

Figure S11. MS (ESI+) Spectra of compound **5g**

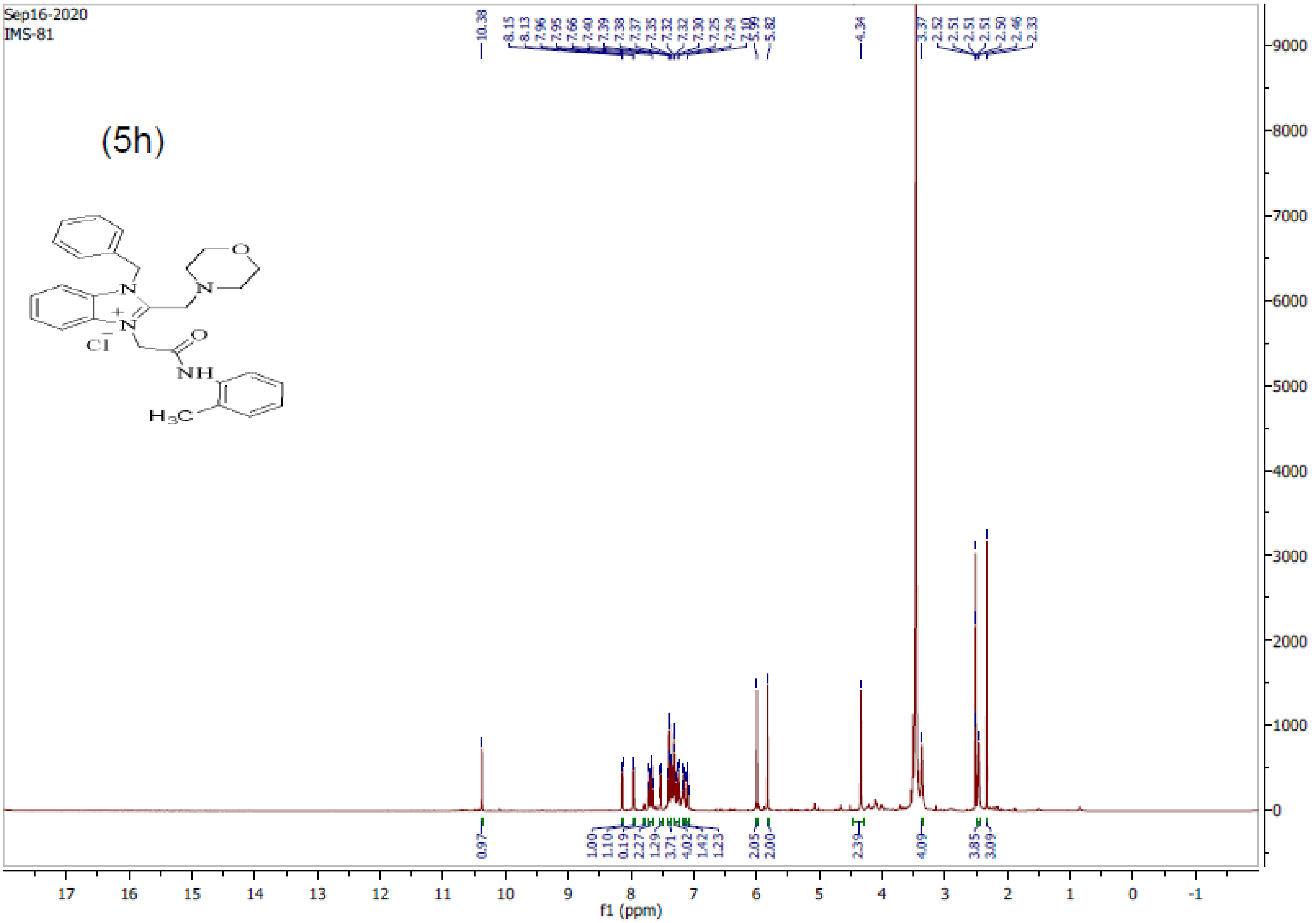

Figure S12. ^{1}H NMR Spectra of compound **5h**

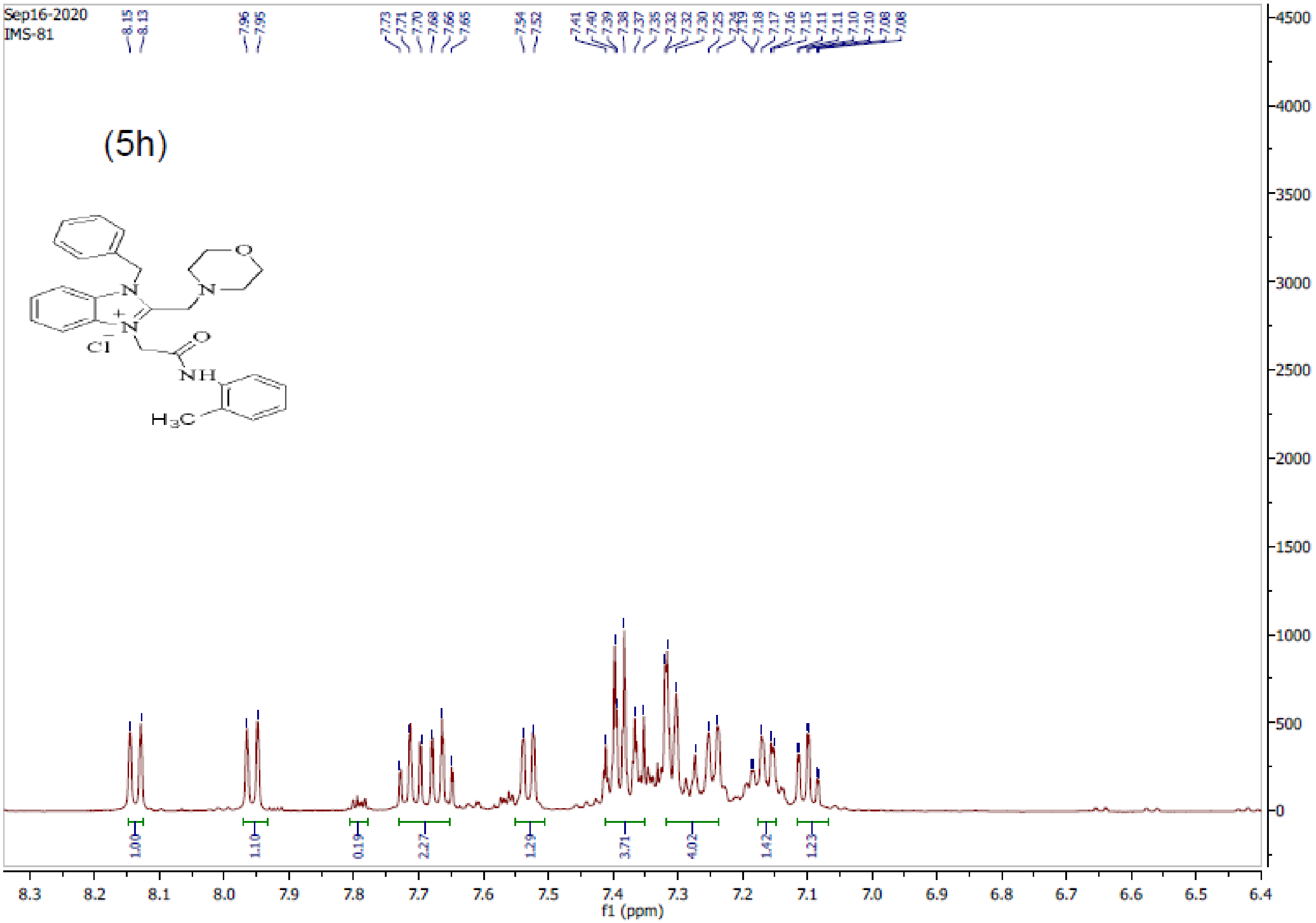

Figure S12a. Expanded version of ^{1}H NMR Spectra of compound **5h**

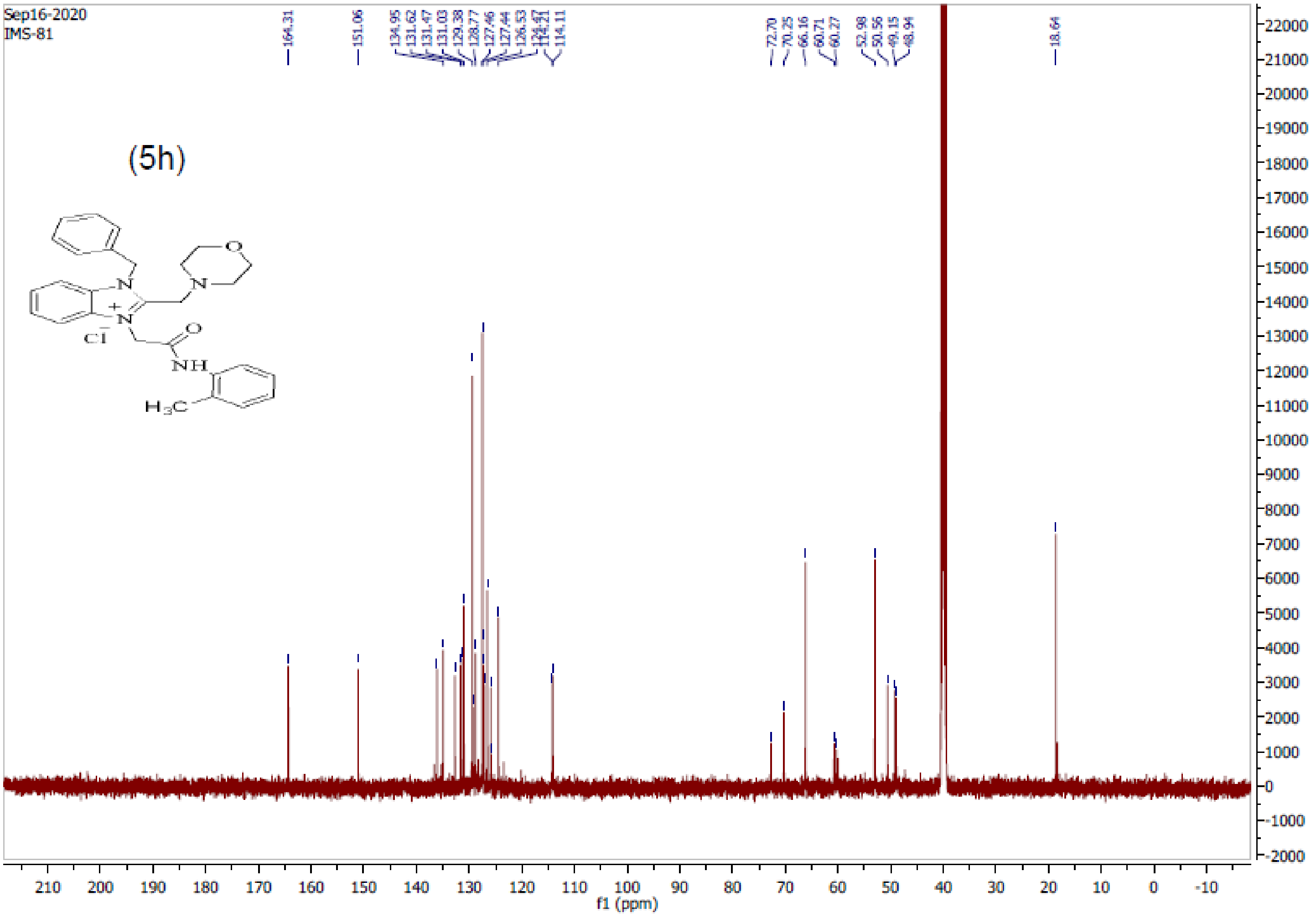

Figure S13. ^{13}C NMR Spectra of compound **5h**

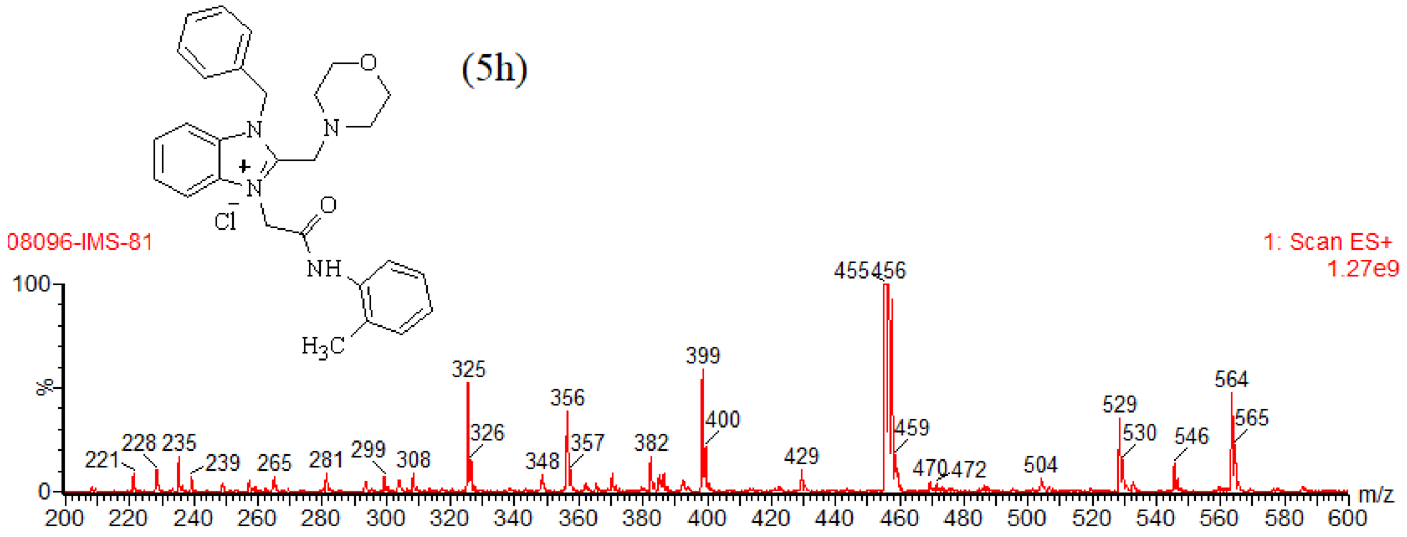

Figure S14. MS (ESI+) Spectra of compound **5h**

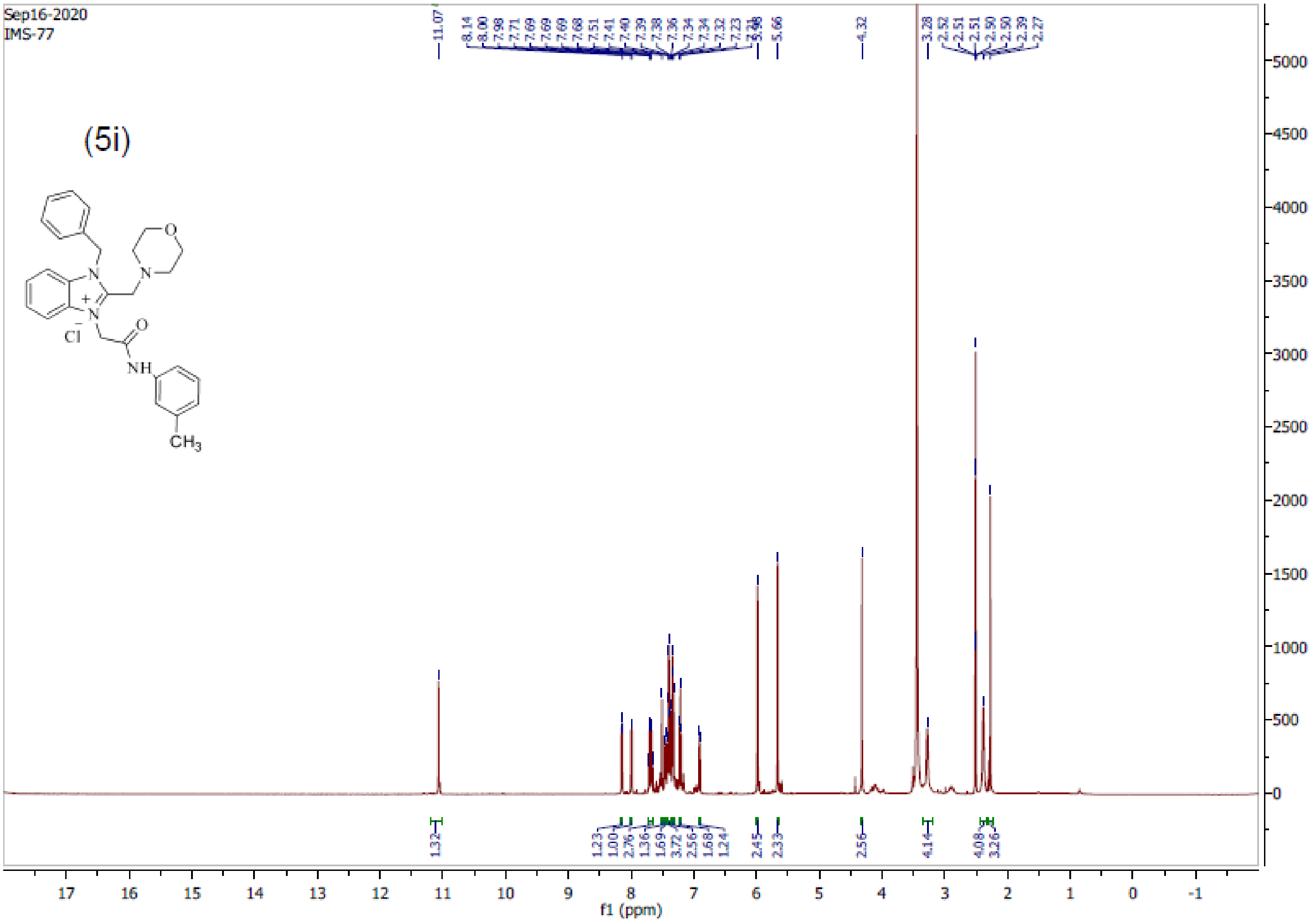

Figure S15. ^{1}H NMR Spectra of compound **5i**

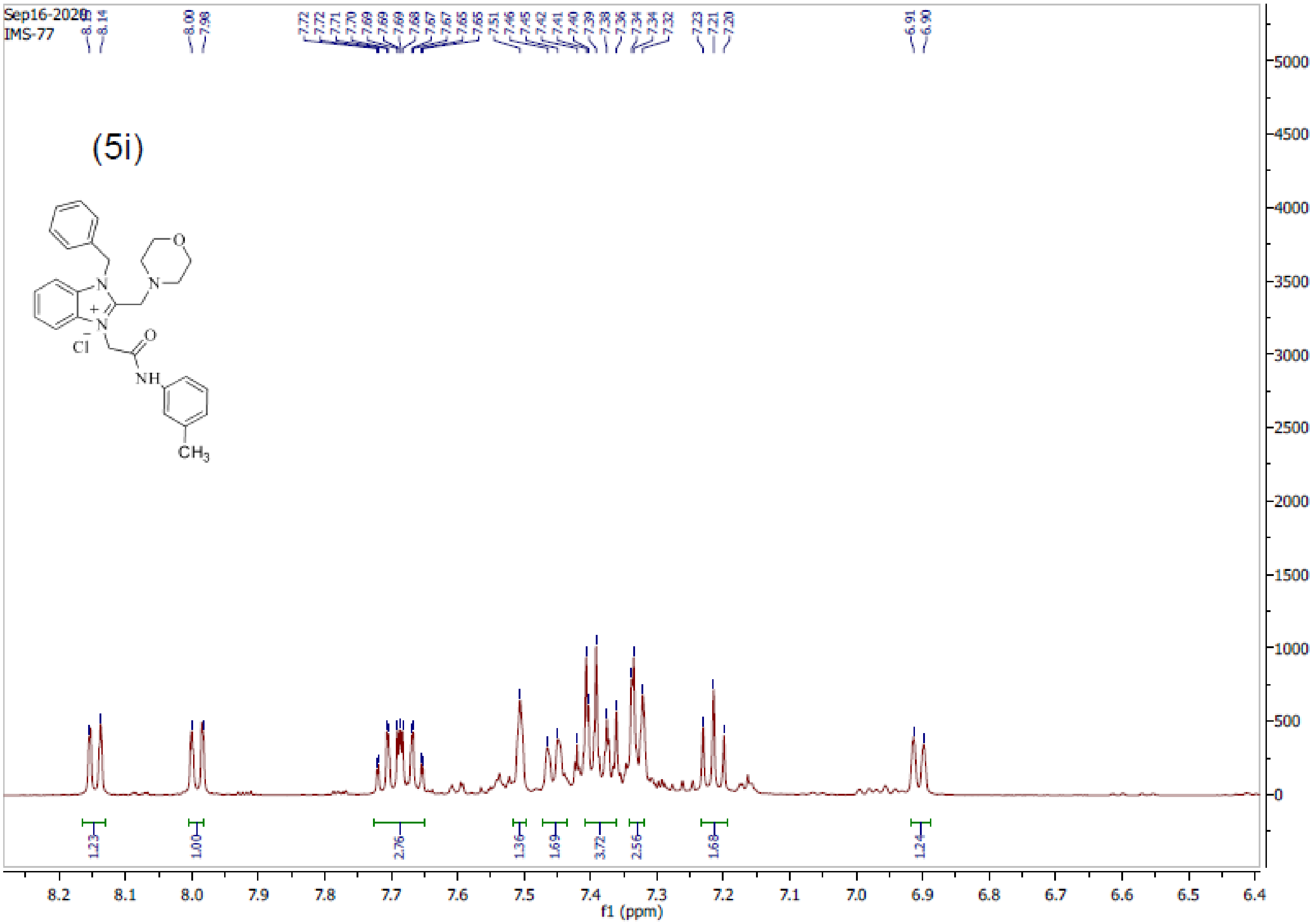

Figure S15a. Expanded version of ^{1}H NMR Spectra of compound **5i**

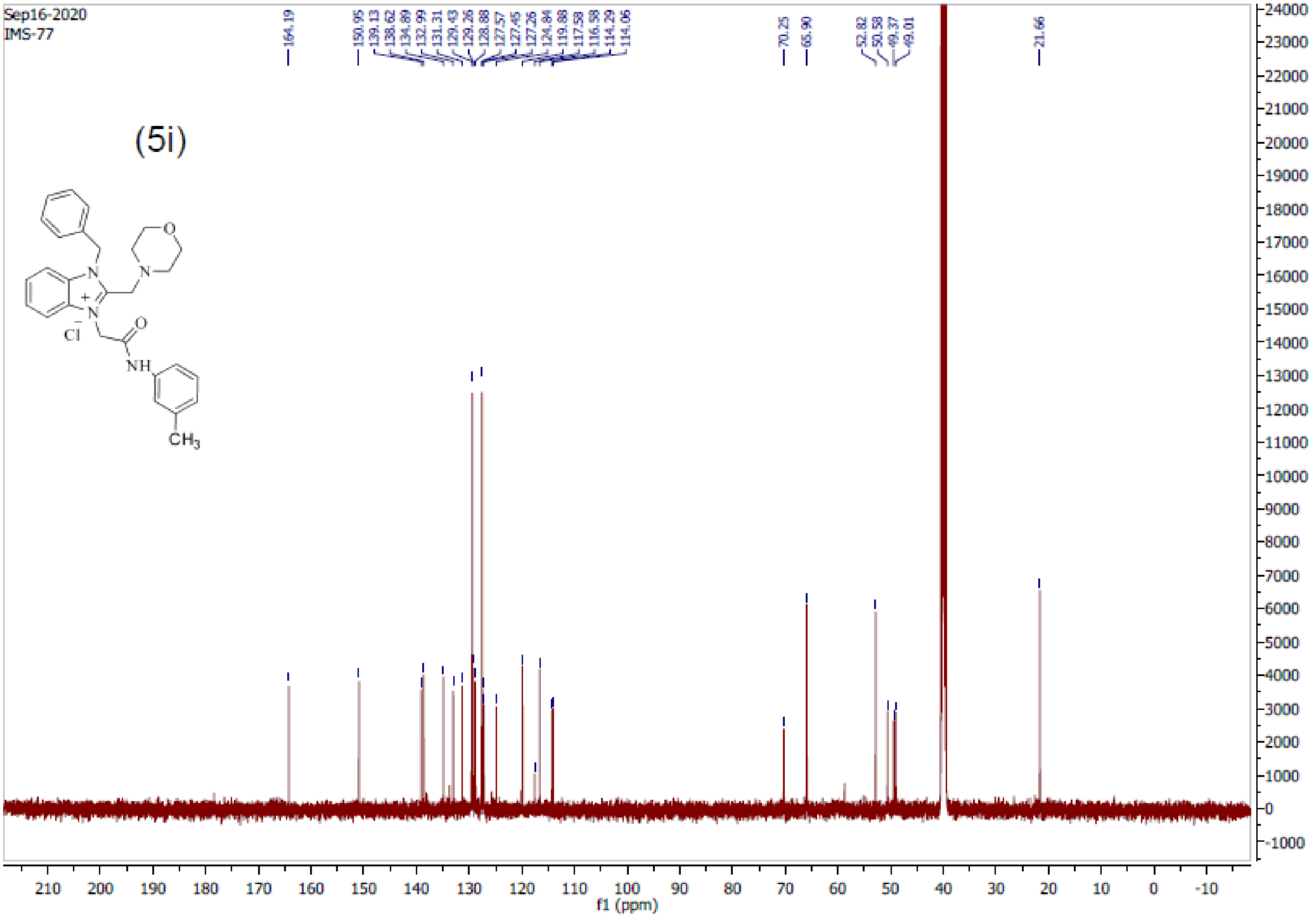

Figure S16. ^{13}C NMR Spectra of compound **5i**

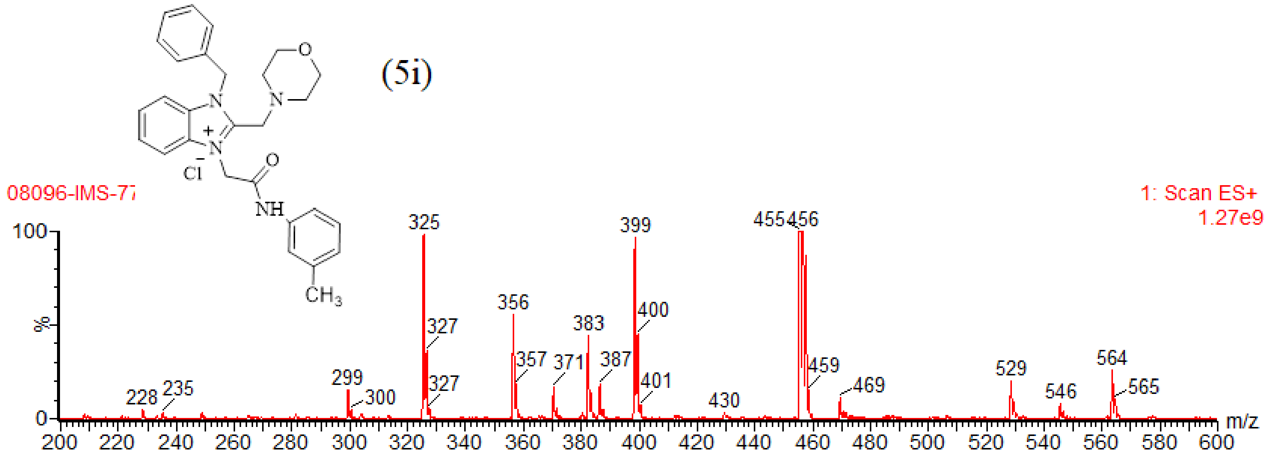

Figure S17. MS (ESI+) Spectra of compound **5i**

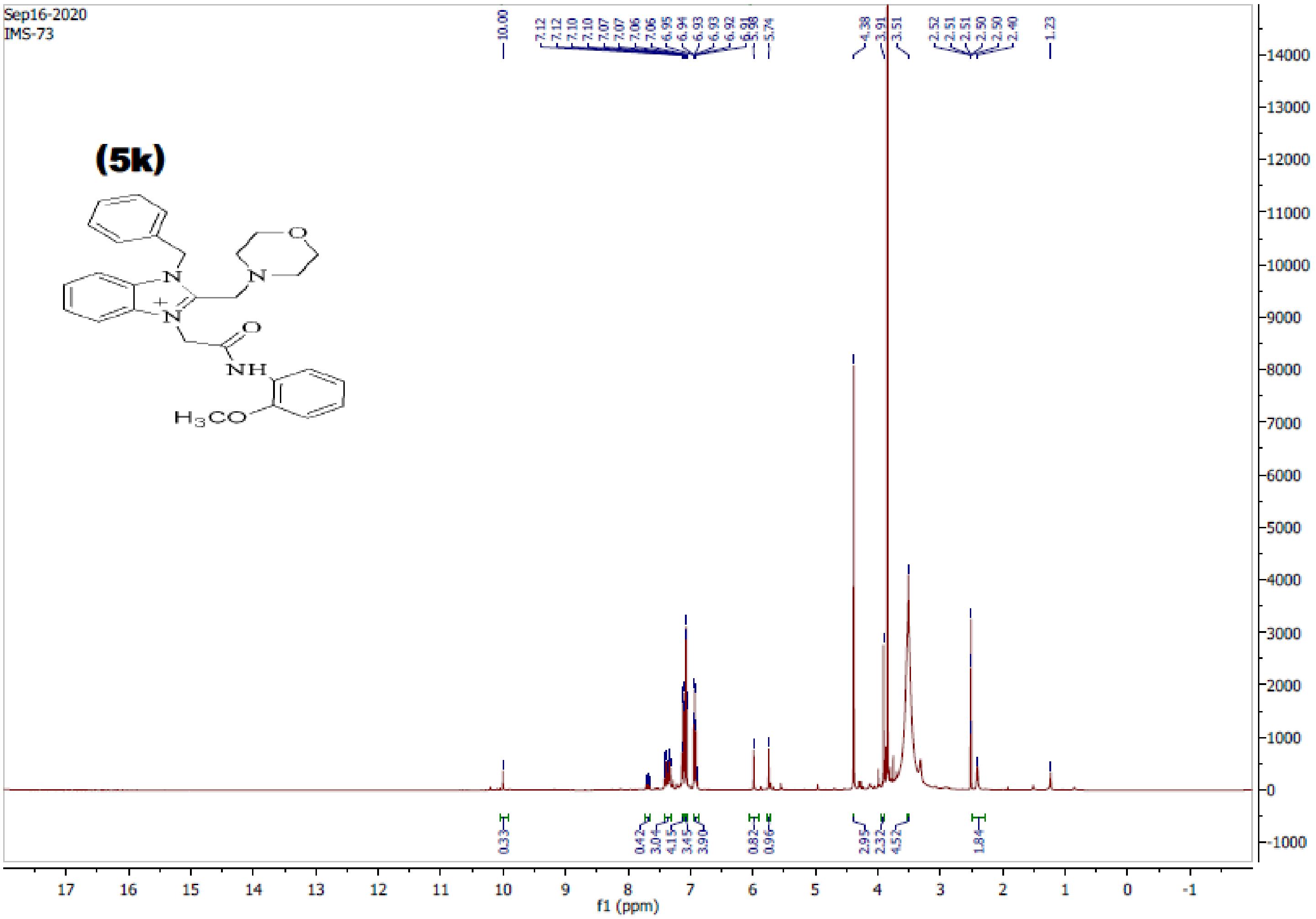

Figure S18. ^{1}H NMR Spectra of compound **5k**

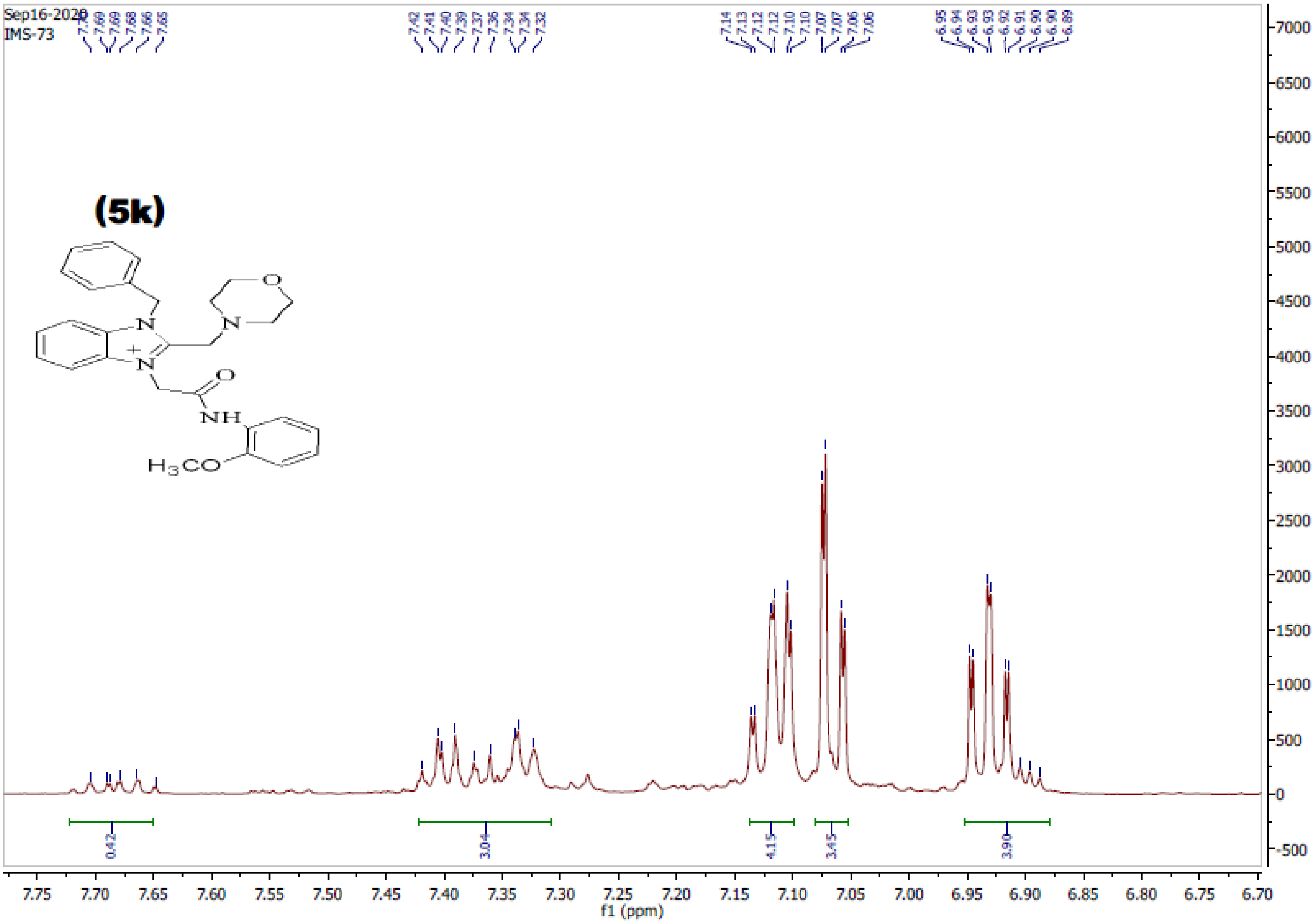

Figure S18a. Expanded version of ^{1}H NMR Spectra of compound **5k**

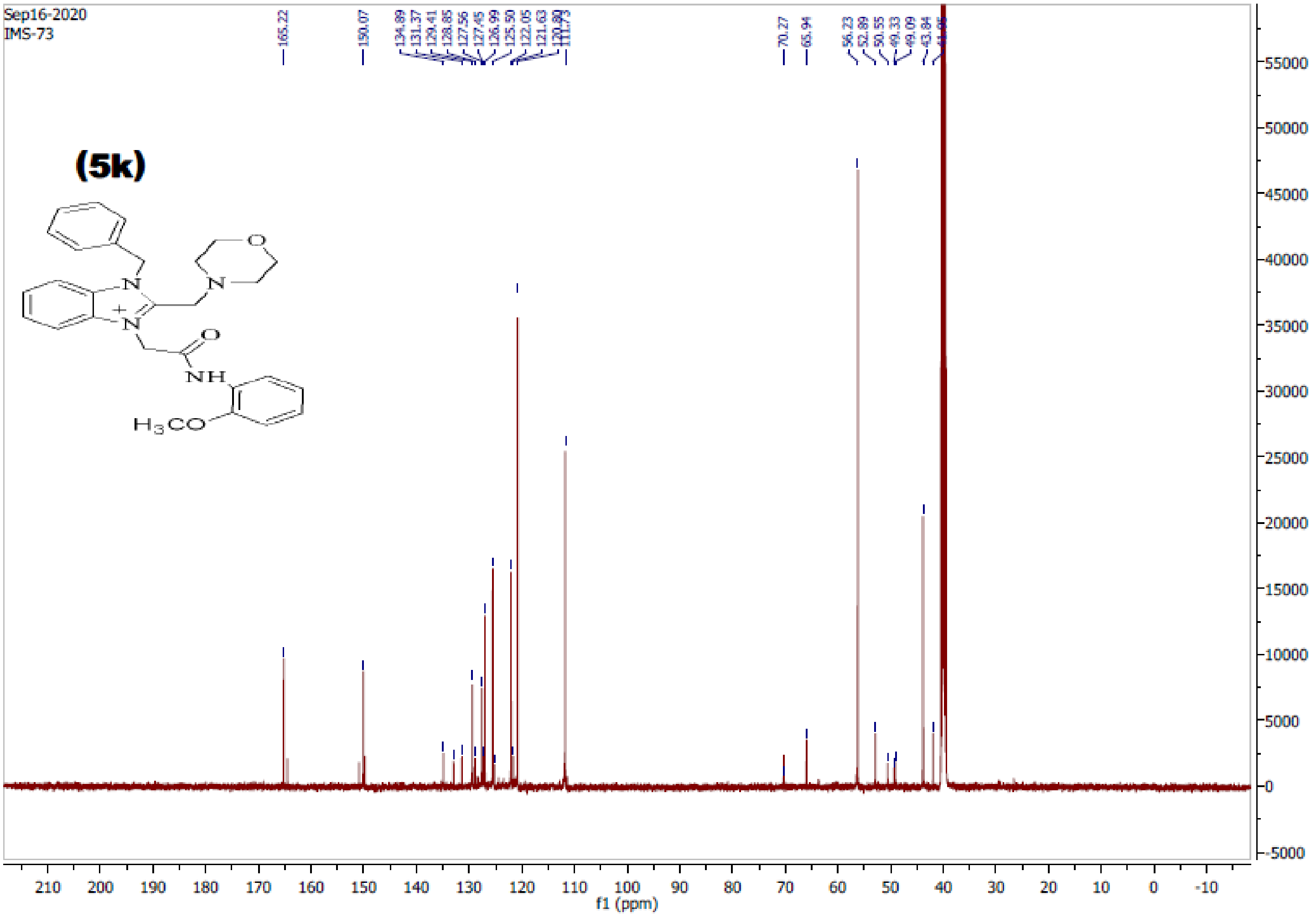

Figure S19. ^{13}C NMR Spectra of compound **5k**

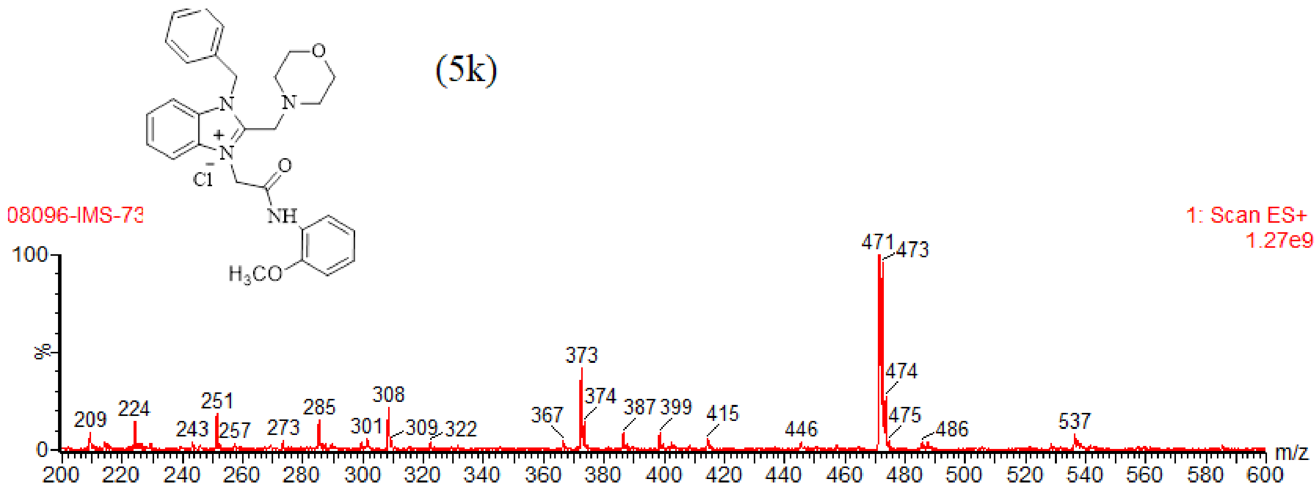

Figure S20. MS (ESI+) Spectra of compound **5k**

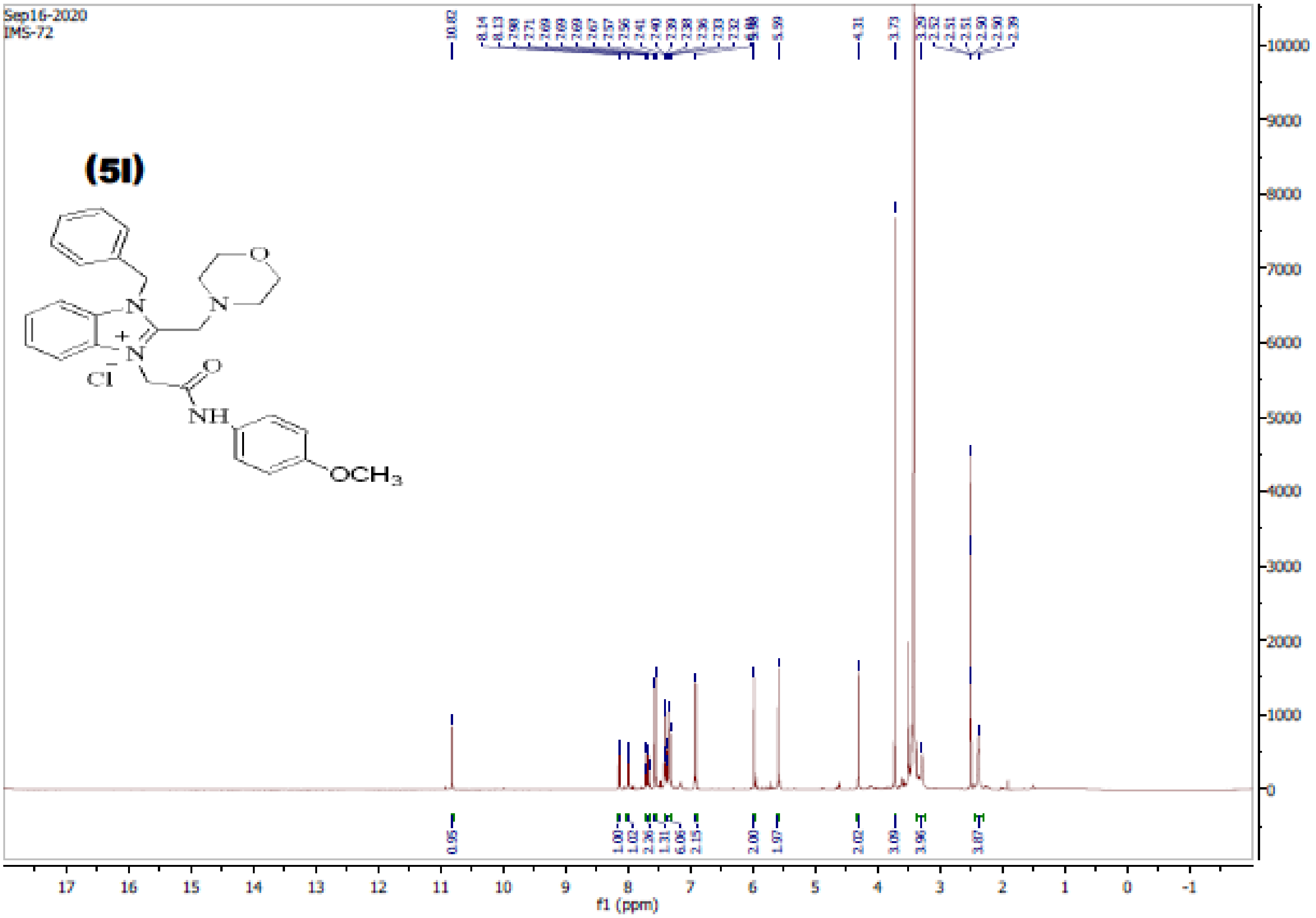

Figure S21. ^{1}H NMR Spectra of compound **5l**

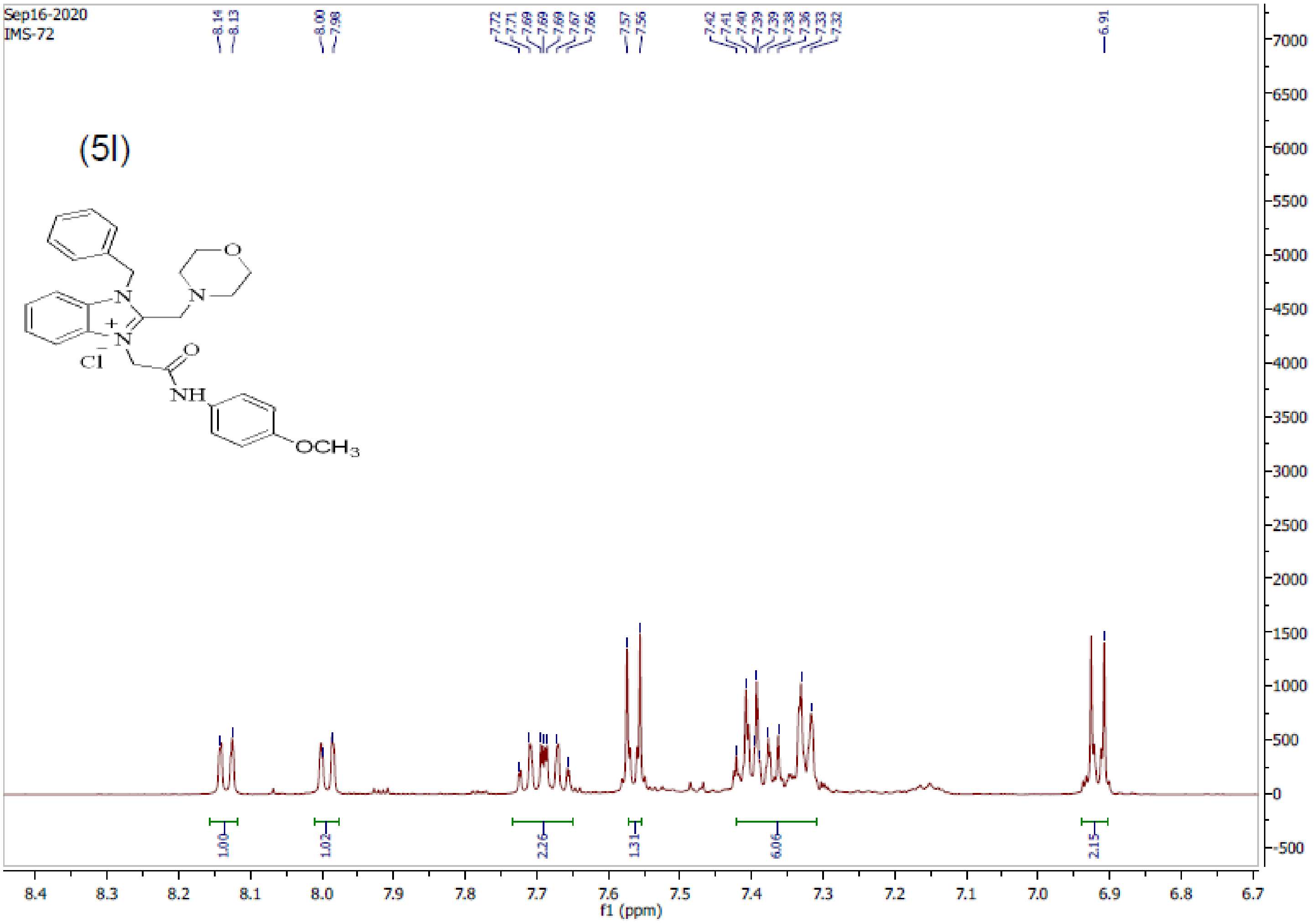

Figure S21a. Expanded version of ^{1}H NMR Spectra of compound **5l**

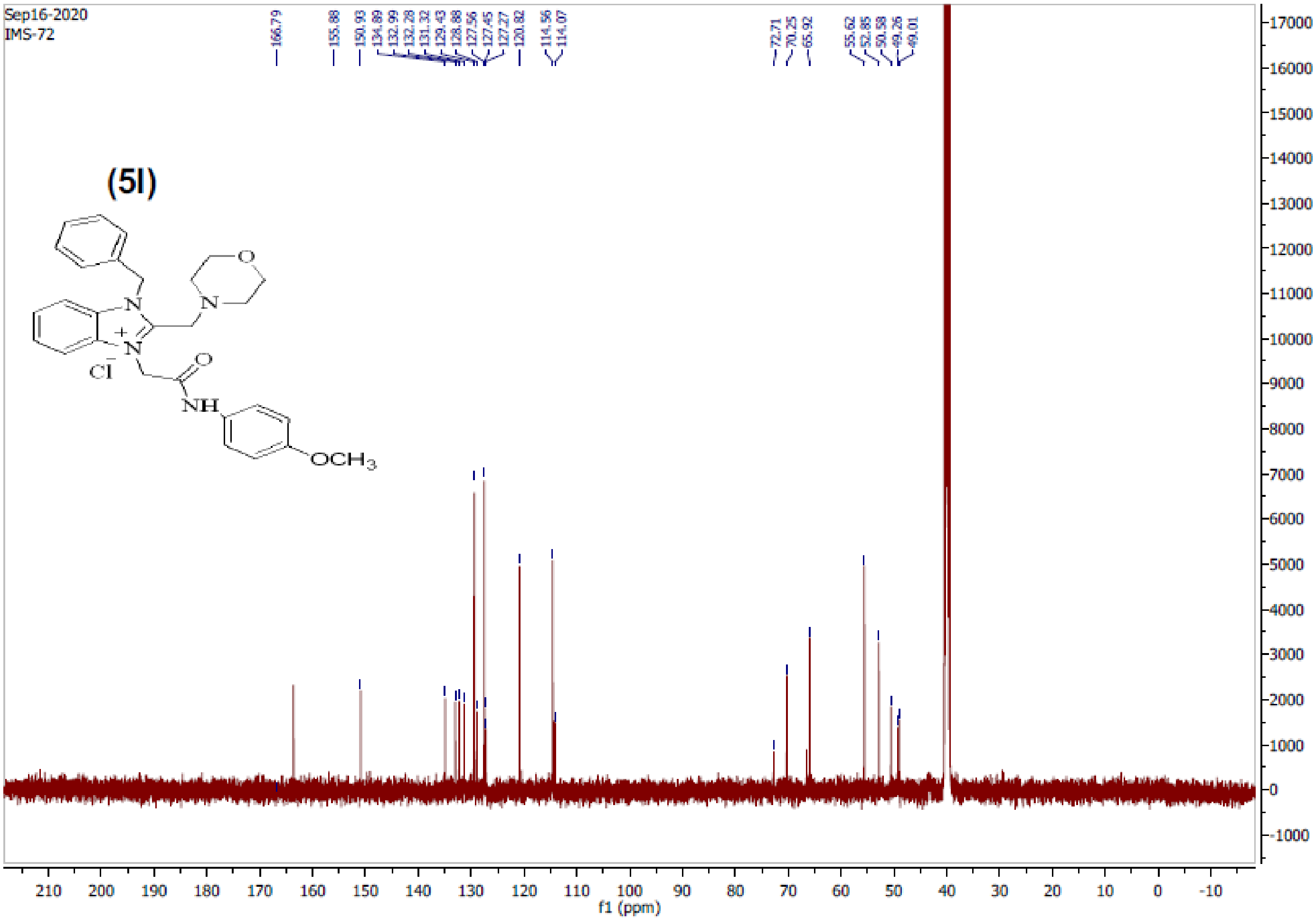

Figure S22. ^{13}C NMR Spectra of compound **5l**

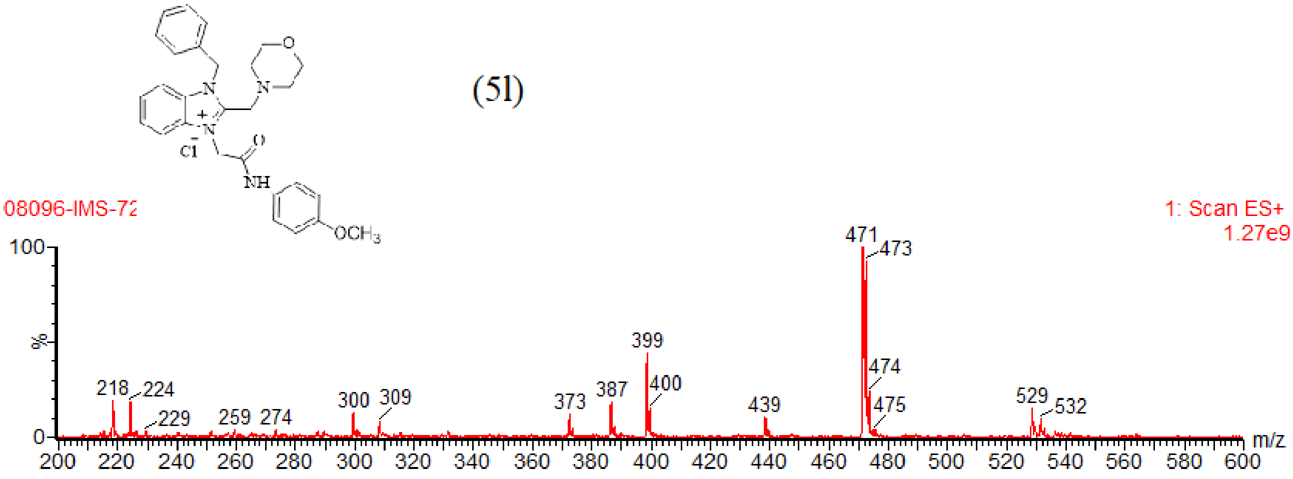

Figure S23. MS (ESI+) Spectra of compound **5l**

Docking Result File (PDB)

Database Viewer : ~/downloads/docking results file.mdb

File Edit Display Compute Window Help Cancel

	mol	rseq	mseq	S	rmsd_refine	E_conf	E_place	E_score1	E_refine	E_score2
1	5d.cdx	1	1	-13.1522	1.0154	129.0979	-36.6264	-11.8805	-10.6274	-13.1522
2	5d.cdx	1	1	-10.6085	2.2935	121.8975	-72.8923	-11.9782	-19.1738	-10.6085
3	5d.cdx	1	1	-10.3569	2.0466	123.1545	-76.5811	-13.2395	-19.0019	-10.3569
4	5d.cdx	1	1	-10.2890	3.9102	127.8784	-56.9124	-12.3152	-16.1065	-10.2890
5	5d.cdx	1	1	-10.1278	2.1802	125.7023	-63.1927	-12.0586	-14.5824	-10.1278
6	5d.cdx	1	1	-10.0827	2.4374	119.7514	-89.9797	-13.1810	-18.6580	-10.0827
7	5d.cdx	1	1	-9.5954	2.3931	121.3401	-91.8234	-12.6033	-14.7198	-9.5954
8	5f.cdx	1	2	-13.1097	1.1559	138.4252	-94.9978	-12.9638	-10.6402	-13.1097
9	5f.cdx	1	2	-12.8787	1.5859	136.0739	-83.9786	-13.2120	-22.4568	-12.8787
10	5f.cdx	1	2	-11.8378	1.6872	142.8584	-76.1641	-13.2848	-10.1926	-11.8378
11	5f.cdx	1	2	-11.4989	2.3017	140.8121	-46.6304	-12.5458	-16.9857	-11.4989
12	5f.cdx	1	2	-11.4962	1.9405	144.8446	-96.2359	-12.4695	-17.2907	-11.4962
13	5f.cdx	1	2	-11.4506	1.3227	137.9221	-112.7603	-12.5989	-12.5161	-11.4506
14	5f.cdx	1	2	-11.3162	2.6433	150.2254	-100.6673	-13.8101	-19.4411	-11.3162
15	5f.cdx	1	2	-11.1075	1.4384	149.5807	-67.4070	-12.4908	-5.9708	-11.1075
16	5f.cdx	1	2	-11.0843	3.0299	138.6966	-73.2426	-12.6254	-10.2588	-11.0843
17	5f.cdx	1	2	-9.7260	2.2931	139.9807	-54.5668	-12.5532	-12.0665	-9.7260
18	5g.sdf	1	3	-12.1094	1.4519	152.3078	-62.5990	-12.1932	-21.9187	-12.1094
19	5g.sdf	1	3	-11.1984	1.0402	153.0056	-87.6709	-13.2276	-23.4009	-11.1984
20	5g.sdf	1	3	-11.1440	2.3871	153.9131	-79.7020	-12.6995	-17.5909	-11.1440
21	5g.sdf	1	3	-11.1024	1.8567	161.3698	-89.4236	-12.3566	-11.7693	-11.3924
22	5g.sdf	1	3	-10.8348	2.5212	153.4076	-62.8999	-13.4171	-22.2705	-10.8348
23	5g.sdf	1	3	-10.6580	2.1049	155.0633	-87.2942	-12.3307	-22.2517	-10.6580
24	5g.sdf	1	3	-10.4493	2.7214	162.6926	-52.6545	-12.2741	-18.5479	-10.4493
25	5g.sdf	1	3	-10.4015	2.7450	159.4362	-45.3219	-12.3387	-15.2879	-10.4015
26	5g.sdf	1	3	-10.2891	1.2385	156.8586	-65.9758	-12.9261	-12.6365	-10.2891
27	5g.sdf	1	3	-9.7101	2.8760	153.2682	-76.4912	-14.0774	-14.7460	-9.7101
28	5h.cdx	1	4	-12.7556	1.1945	138.9458	-76.1925	-12.6886	-22.6892	-12.7556
29	5h.cdx	1	4	-11.9848	1.4645	148.3145	-51.9860	-11.8850	-18.6671	-11.9848
30	5h.cdx	1	4	-11.1968	1.1108	149.7834	-104.9779	-12.1762	-14.2573	-11.1968
31	5h.cdx	1	4	-10.9013	2.7360	138.0410	-35.6136	-12.7254	-20.4680	-10.9013
32	5h.cdx	1	4	-10.7550	1.8702	138.3940	-66.1726	-12.3945	-20.7853	-10.7550
33	5h.cdx	1	4	-10.4373	1.4958	137.9394	-86.0175	-11.8923	-23.8529	-10.4373
34	5h.cdx	1	4	-10.2923	1.9672	139.5539	-80.6186	-12.8072	-21.1069	-10.2923
35	5k.cdx	1	5	-12.1551	1.3817	140.9769	-84.5263	-11.9644	-16.6682	-12.1551
36	5k.cdx	1	5	-11.5357	1.2696	141.1774	-79.7806	-11.9910	-20.7532	-11.5357
37	5k.cdx	1	5	-11.1027	2.5555	144.3948	-79.5311	-14.0631	-19.2903	-11.1027
38	5k.cdx	1	5	-10.6015	1.6184	140.5226	-73.3014	-11.5994	-18.4611	-10.6015
39	5k.cdx	1	5	-10.5323	2.6144	142.8769	-30.8938	-11.7920	-19.5241	-10.5323
40	5k.cdx	1	5	-10.3643	2.5998	148.4182	-82.5180	-13.8225	-21.7493	-10.3643
41	5k.cdx	1	5	-10.1677	2.1209	144.2799	-79.5934	-11.5327	-18.1309	-10.1677
42	5k.cdx	1	5	-10.0046	1.9708	139.4478	-63.7198	-11.4900	-21.1347	-10.0046
43	5k.cdx	1	5	-9.7639	2.6793	144.8671	-60.4646	-12.1559	-17.8163	-9.7639
